# Nonmonotonic Charging, Overscreening, and Ion Competition in the Electric Double Layer of Platinum Quantified by X‑ray Spectroscopy

**DOI:** 10.1021/jacs.6c14786

**Published:** 2026-09-26

**Authors:** Alfred Larsson, Dogukan Yilmaz, Jan Sebastian Dominic Rodriguez, Tarik Zahzah, de Haij Pieter, Jordy J.J. Eggebeen, Karen van den Akker, Damien Roy, Solenn Reguer, Marc T.M. Koper, Rik Mom

**Affiliations:** † Leiden Institute of Chemistry, Leiden University, PO Box 9502, Leiden 2300 RA, The Netherlands; ‡ Department of Physics, Tamkang University, New Taipei City 251301, Taiwan; § Synchrotron Soleil, L’Orme des Merisiers, départementale 128, Saint-Aubin 91190, France

## Abstract

The electric double layer (EDL) is an integral part of the local reaction environment in electrocatalysis. Indeed, the strong electric fields and modified interfacial water structure accompanying ion accumulation in the EDL have been shown to impact the performance of nearly every electrocatalytic reaction. However, quantifying interfacial ion accumulation experimentally is a long-standing challenge, leaving the atomic-level picture of electrolyte effects in electrocatalysis incomplete. Here, we address this challenge using an interface-sensitive *in situ* X-ray fluorescence spectroscopy approach based on a mesoporous Pt electrode to track interfacial ion concentration at the Pt-electrolyte interface in acidic media. We show that the EDL structure of the Pt electrode has a complex dependence on the applied potential and electrolyte composition. From a systematic analysis of ten electrolytes (including ions such as ClO_4_^–^, SO_4_^2–^, K^+^, and Ca^+^), we show that adsorbates alter the surface free charge, resulting in nonmonotonic charging and overscreening. For nonadsorbing anions, we show that the concentration-dependent surface free charge follows classical Gouy–Chapman theory. At negatively charged surfaces, cations and protons compete to screen the surface free charge in the EDL, with a strong dependence on ion valency.

## Graphical abstract

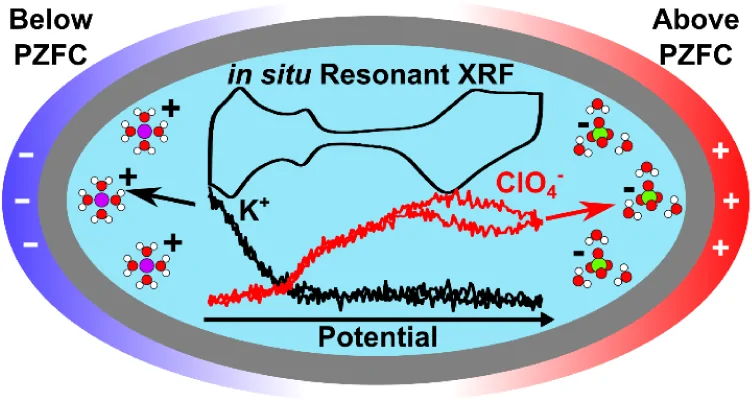

## Introduction

Electrochemical reactions occur at the interface between an electronic conductor and an electrolyte, where an electric double layer (EDL) forms. Such EDL formation includes interfacial ion accumulation, strong local electric fields, and reorganization of solvent molecules, which directly influence reaction thermodynamics and kinetics in electrocatalysis.^1–6^ Resolving the atomic structure of the EDL and its response to electrode potential and electrolyte composition is therefore a central challenge in electrochemistry.

Classical descriptions of the EDL, based on the Gouy–Chapman–Stern (GCS) framework,^7^ assume that the electrochemical interface is ideally polarizable i.e., free of adsorbates with charge transfer to the electrode. These assumptions are often not valid, however, even for well-defined model systems.^8,9^ On metal electrodes such as platinum, the surface is rarely free of adsorbates, even within the so-called double-layer region.^10^ Adsorbed species such as hydrogen, hydroxide (OH),^11,12^ and specifically adsorbing anions modify the interfacial electronic structure and thus the electrostatic boundary conditions experienced by the electrolyte.^13^ Moreover, electrocatalysis is typically performed in concentrated electrolytes and in the presence of adsorbates, where classical GCS theory is no longer applicable. Since there is no established quantitative EDL theory for these conditions, it is essential to determine experimentally how system-specific factors, such as the type and coverage of adsorbates, ion sizes, and interfacial dielectric behavior, influence EDL structure.

Our current experimental understanding of the EDL is largely based on differential capacitance measurements, as the technique is experimentally straightforward and the capacitance can be directly compared to classical models.^14,15^ However, such measurements are restricted to narrow potential windows free of adsorbates and pseudocapacitive contributions. For reactive metals such as Pt, this corresponds to a ∼200 mV window on Pt(111) in which anomalously low electrolyte concentrations are required to observe the Gouy–Chapman predicted capacitance minima at the potential of zero free charge (PZFC).^8^ Consequently, experimental insight into the adsorbate-free EDL using capacitance measurements is largely limited to conditions where classical behavior applies.

Accessing the EDL under catalytically relevant conditions, therefore, requires alternative experimental approaches.^16,17^ Many efforts have been made, including the radioactive tracer method,^18,19^ scanning probe microscopy,^20^ surface X-ray diffraction,^21,22^ vibrational spectroscopy,^23,24^ and X-ray spectroscopy.^25,26^ However, techniques that allow direct, straightforward, and quantitative probing of ions in the EDL are still lacking.

Here, we combine cyclic voltammetry with *in situ* resonant X-ray fluorescence spectroscopy (XRF) on mesoporous Pt electrodes. The large interface-to-bulk electrolyte ratio within the porous electrode significantly enhances the spectroscopic signal from the solid–liquid interface. Moreover, we use one of the most studied model systems in electrochemistry: platinum in acidic media. This system is not only highly relevant to catalysis but also provides a range of adsorbates across the potential window, with regions of both anion- and cation-dominated charge screening, allowing us to deliberately study how adsorbates influence the EDL.

## Results and Discussion

To directly probe interfacial ion concentrations, we use *in situ* resonant XRF during cyclic voltammetry of a mesoporous (platinum) electrode. The electrochemical flow cell used for the experiment is shown in Figure 1(a), where the working electrode, consisting of mesoporous Pt electrodeposited on a Au-coated SiN_*x*_ membrane, is highlighted in (b). For the tender X-rays used here, the X-ray beam entering the cell is largely attenuated by the mesoporous Pt film, so that primarily the electrolyte within the mesopores is probed. This induces interface sensitivity in the measurements, as the ratio of interfacial to bulk electrolyte is large within the pores, as illustrated (approximately) to scale in Figure 1(c), based on scanning electron microscopy (SEM) analysis of the pore structure (Figure 1(d) and Supporting Information Section 1). The diameter of the mesopores is ∼7 nm, which is larger than the Debye length for the ionic strengths used in this work. Hence, there is no significant double layer overlap or confinement effects due to the nature of the porous electrode (further discussed in the Supporting Information Section 2). Furthermore, care was taken to avoid mass-transfer limitations or potential gradients across the mesoporous film, as further discussed in the Supporting Information Sections 3 and 4. These considerations are important, as they facilitate translation of our results using mesoporous Pt to commonly used flat polycrystalline Pt electrodes.

**Figure 1:**
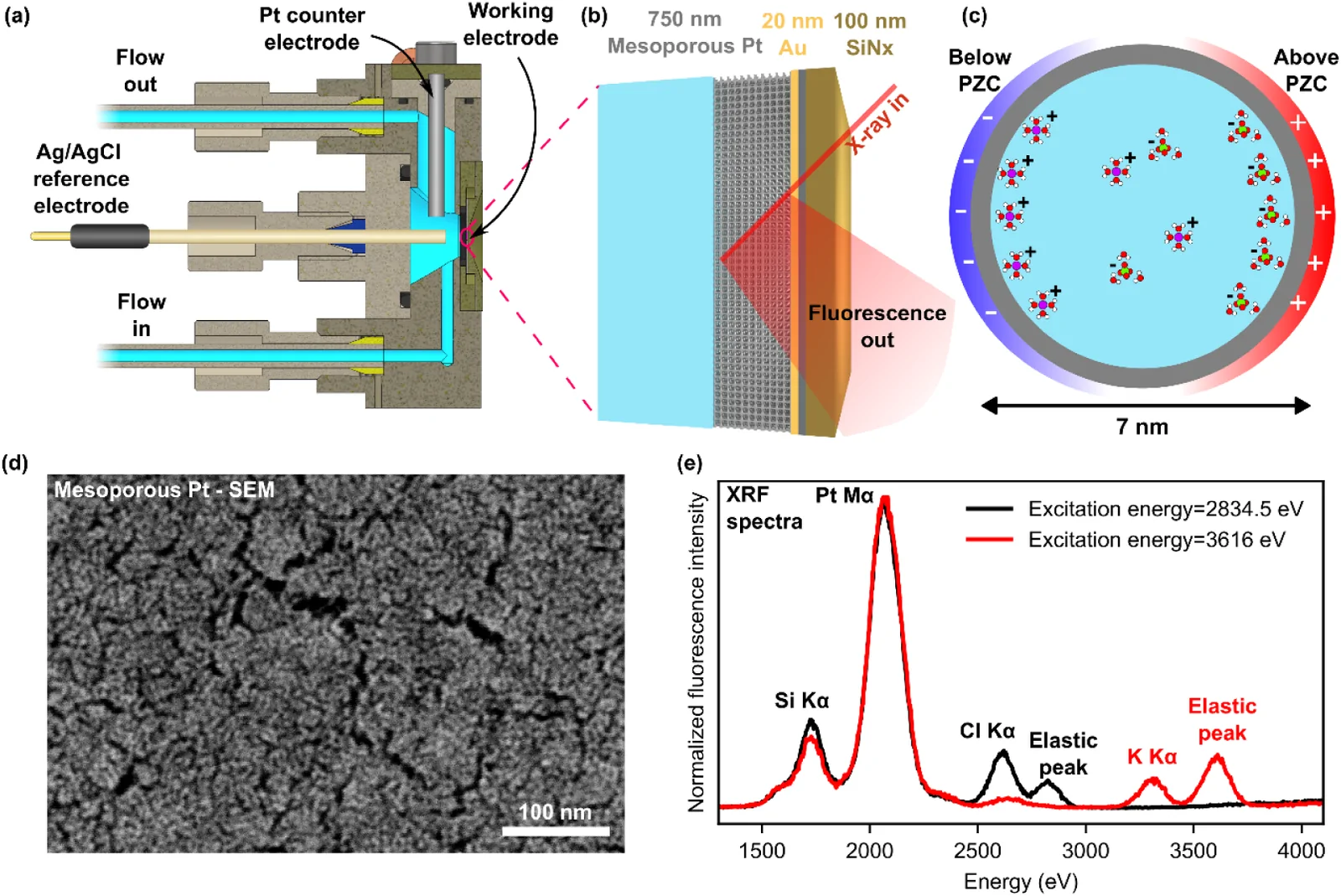
(a) Cross-sectional view of the electrochemical flow cell for *in situ* resonant XRF experiments. (b) Schematic illustration of the sample and measurement geometry. (c) Schematic view of a pore with hydrated ions drawn (approximately) to scale. (d) Top-view SEM image of the mesoporous Pt electrode. (e) XRF spectra acquired at different excitation energies used to probe the Cl Kα and K Kα fluorescence lines (Kβ lines are hidden in the elastic peaks).

Figure 1(e) shows two examples of XRF spectra, recorded in resonance with the Cl K-edge or K K-edge whiteline in 10 mM HClO_4_ + 25 mM KClO_4_ in a 1 s time interval. The spectra show the elements from the sample (Si from the SiN_*x*_ window and Pt from the mesoporous electrode) as well as the ions in the electrolyte (Cl from ClO_4_^–^ and K from K^+^). The high Cl Kα signal at 2834.5 eV is caused by resonant excitation (higher cross section), which maximizes the signal, as illustrated in the Supporting Information, Figure S11. The same strategy to achieve high XRF signals through resonant excitation was used for all ions. Due to the absence of self-absorption effects in this measurement geometry (discussed in Supporting Information Section 5), Cl Kα and K Kα signals are proportional to the interfacial ClO_4_^–^ and K^+^ concentration. Exploiting this, together with the high time resolution of the methodology, we have used the *in situ* resonant XRF to track the interfacial concentration of ClO_4_^–^, SO_4_^2–^, K^+^ and Ca^2+^ ions as a function of potential during cyclic voltammetry. The reproducibility of the signals is illustrated in the Supporting Information Section 12.

Figure 2(a) shows a cyclic voltammogram of the mesoporous Pt electrode in 10 mM HClO_4_ + 25 mM KClO_4_ together with the corresponding resonant XRF signal of K^+^ and ClO_4_^–^. Note that the voltammograms exhibit all the expected features of polycrystalline Pt in acid, including the hydrogen underpotential deposition (H_UPD_) region (0.05–0.4 V), the double-layer region (0.4–0.8 V), and the onset of surface oxidation above 0.8 V.^27^ Also on the quantitative level, the shapes and positions of these features match those observed on planar Pt electrodes, highlighting that our results obtained with mesoporous Pt electrodes can be generalized to planar polycrystalline Pt interfaces. The corresponding evolution of the adsorbate structure over the entire potential range is schematically illustrated in Figure 2(c). Importantly, even within the so-called double-layer region, the surface is not free of adsorbates, as OH adsorption on step sites already occurs within the H_UPD_ region.^11,12,28–30^

**Figure 2:**
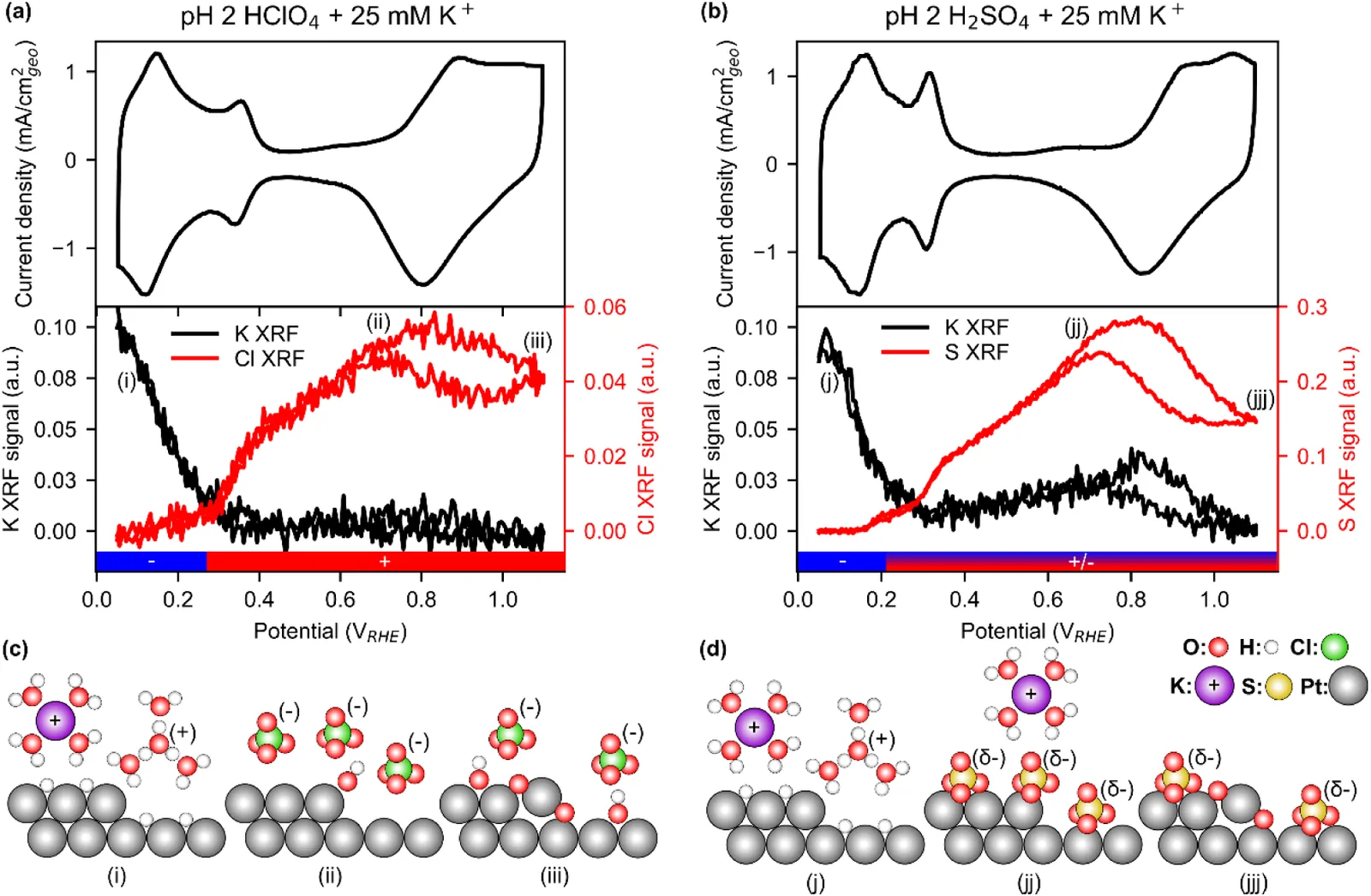
(a) (top panel) Cyclic voltammogram in 10 mM HClO_4_ + 25 mM KClO_4_ measured at 10 mV/s in the flow cell while simultaneous XRF was acquired *in situ*. (bottom panel) XRF intensities of the K and Cl Kα as a function of electrode potential during cyclic voltammetry. (b) (top panel) Cyclic voltammogram in 10 mM H_2_SO_4_ + 12.5 mM K_2_SO_4_ measured at 10 mV/s in the flow cell while simultaneous XRF was acquired. (bottom panel) XRF intensities of the K and S Kα as a function of electrode potential during cyclic voltammetry. (c, d) Atomistic schematics of the interface at selected potentials indicated in (a) and (b), respectively.

The bottom panel of Figure 2a shows the corresponding XRF traces, in which the bulk contribution has been subtracted to obtain the interfacial ion signal within the EDL (subtraction procedure is described in more detail in the Supporting Information Section 13). The extracted interfacial ion distributions reveal a clear potential-dependent transition in the dominant ionic species, while also highlighting deviations from simple monotonic charging behavior. At low potentials, the EDL is dominated by cations (K^+^), whereas at higher potentials it is dominated by anions (ClO_4_^–^), as schematically illustrated in Figure 2(c). Outside a narrow transition region around 0.28 V_RHE_, a single ion species dominates the interfacial electrolyte, indicating the absence of significant cation–anion pairing in this nonspecifically adsorbing electrolyte. This is also confirmed using complementary electrochemical quartz crystal microbalance (EQCM) measurements (Supporting Information Section 6, Figure S5), illustrating that the observed effects are not induced by the X-ray beam.

The crossover between cation and anion accumulation around 0.28 V_RHE_ identifies the potential at which the dominant interfacial ion signal (positive Gibbs excess) switches from cations to anions. This potential is closely associated with the PZFC, where the sign of the surface free charge changes from negative to positive. However, because the XRF measurement probes the interfacial ion signals of K^+^ and ClO_4_^–^ with different unknown calibration factors, the exact value of the PZFC cannot be identified directly from the data. In addition, as can be expected for the polycrystalline Pt employed here, the transition from cation to anion dominated EDL is not sharp, and a narrow potential window exists in which both cations and anions are detected. Such coexistence occurs even for ideally polarizable, structurally homogeneous electrodes (e.g., Hg in NaCl electrolyte^31^), but is strengthened here due to the lateral heterogeneity of surface sites on polycrystalline Pt, which exhibit slightly different adsorbate energetics and therefore different local PZFCs.^32,33^

A further complexity in the PZFC behavior is identified when analyzing the pH dependence of the onset potential for cation accumulation. According to classical theory, the PZFC should be constant on the standard hydrogen electrode (SHE) scale and vary with pH on the RHE scale, since the surface charging does not involve protons. However, this is not observed here, as shown in the Supporting Information Section 7. Instead, the potential at which cation attraction starts remains approximately constant at 0.28 V vs RHE and does not exhibit the expected 59 mV/pH shift on the RHE scale. This indicates that the sign change in surface free charge is not governed by a classical, adsorbate-independent PZFC.

Further support for the influence of adsorbates on EDL formation comes from the nonmonotonic trend observed in the interfacial ClO_4_^–^ concentration. Upon entering the oxide formation region, the anion signal decreases despite increasing potential. Underlining that this is directly related to the presence of surface oxides, the anion concentration shows a hysteresis between the forward and backward potential scan, mirroring the irreversibility between Pt surface oxide formation and reduction.

Since the ClO_4_^–^ signal is proportional to the surface free charge when cations are absent in the EDL, as is the case in the potential range for surface oxide formation and reduction, the link between surface oxidation and nonmonotonic ClO_4_^–^ accumulation implies an analogous nonmonotonic trend in the surface free charge. This experimentally observed nonmonotonic change in surface free charge with potential has previously been theoretically described by Huang and Eikerling,^34,35^ following an original discovery by Frumkin et al.,^19^ and reflects a coverage-dependent shift in the PZFC, caused by a shift in the work function associated with surface oxidation.^36,37^ As the oxide coverage increases, the corresponding shift in the PZFC (*E*_*PZCF*_) can outpace the applied electrode potential (*E*), thereby decreasing the difference between the electrode potential and the PZFC, which leads to a lower rational potential (*E*_*Rational*_ *= E* – *E*_*PZFC*_),^31^ as shown in Figure 3 (the curves are intended solely as qualitative illustrations). Consequently, the positive surface free charge can decrease with increasing electrode potential.

**Figure 3:**
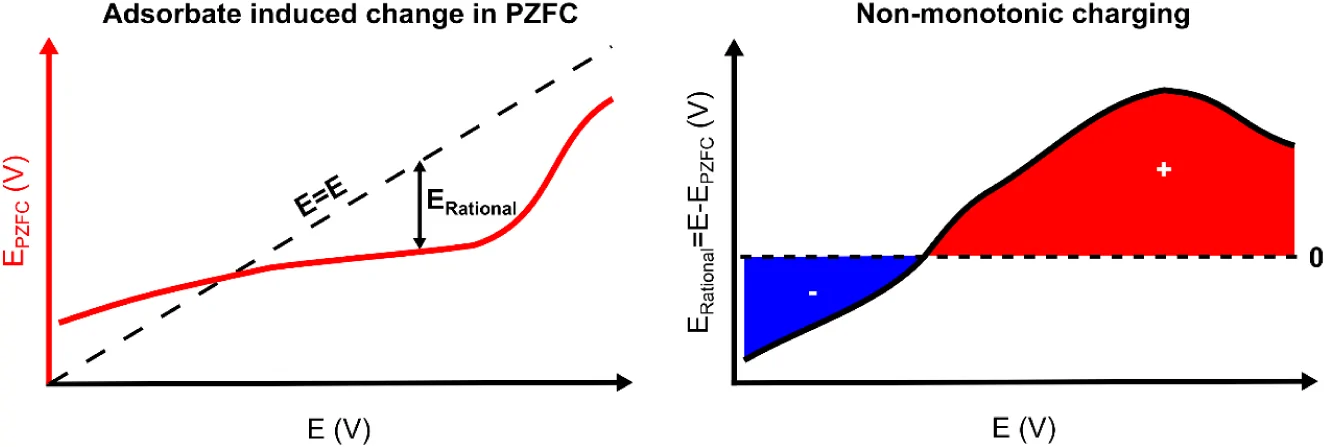
Schematic qualitative illustration of how the shift in PZFC as a function of potential can outpace the applied potential and lead to nonmonotonic charging.

Along similar lines, the nonmonotonic change in surface free charge can also be rationalized by considering the generation of metal–oxygen dipoles during oxidation, which induces an analogous shift in the PZFC.^34,38^ Effectively, these two explanations are two sides of the same coin, but they do provide slightly different insight into the generalizability of the phenomenon of nonmonotonic changes in surface free charge. Given that the oxidation-induced work function/PZFC modification is a general phenomenon,^36,37,39^ one may anticipate that the nonmonotonic charging observed here is generalizable to systems where OH adsorption and surface oxidation occur. The dipole framework further extends this to other systems where strong dipoles are formed (e.g., during surface chlorination). It would thus be of interest to quantitatively assess the dipole-surface charge correlation. However, charge redistribution of the Pt–O bond cannot be assessed within the XRF setup employed here.

Shifting focus to the shape of the potential-dependent cation signals, another indication that adsorbates dictate surface free charge is evident from the change in slope of the cation signal as a function of potential near E = 0 V_RHE_ (see also Figure 5(a) and (b)). Within this potential range, the adsorbate coverage shifts from a mixed H/OH distribution on steps to a purely H-covered state. This can again affect the work function^37^ and hence the surface free charge, causing a change in the slope of the cation charging curve. This is also observed using EQCM (Supporting Information Section 6, Figure S5) and has been seen in earlier radioactive tracer experiments for Na on Pt.^40^ Overall, our analysis of the Pt-(10 mM HClO_4_ + 25 mM KClO_4_) electrolyte interface thus shows that adsorbates play an important role in EDL formation, affecting the EDL charging properties of both anion and cation dominated EDLs.

To investigate how EDL formation is altered by specific anion adsorption (i.e., chemisorption of anions), we also studied EDL formation in 10 mM H_2_SO_4_ + 12.5 mM K_2_SO_4_. As shown in Figure 2(b), the overall voltammetric response resembles that in perchloric acid, yet some notable differences are observed, including a sharpening of the second H_UPD_ feature (at 0.3 V), and a positive shift of the oxide formation region to more positive potentials, consistent with strong anion–surface interactions.

Correspondingly, we also observe a general resemblance in EDL formation compared to perchloric acid, but with a few key differences. Most notably, in contrast to the perchlorate case, the accumulation of sulfate anions at the surface does not lead to the exclusion of cations. Instead, K^+^ remains present in significant concentration even at potentials where sulfate adsorption is substantial, as schematically illustrated in Figure 2(d). The simultaneous accumulation of sulfate and K^+^ is consistent with overscreening associated with specific sulfate adsorption. Related cation–anion coadsorption phenomena have previously been reported for Pt and other electrode materials,^13,40–44^ which shows the generality of this phenomenon also for more weakly interacting surfaces such as Au. Underlying the overscreening is the chemical interaction of the sulfate ions with the electrode surface, which drives the accumulation of an excess amount of sulfate into the EDL, i.e., more sulfate ions than needed to screen the positive Pt surface free charge. As a result, the sulfate anions are only partially discharged,^45^ and the surface, including adsorbed sulfate ions, is charged net negatively, which is compensated by the electrostatic attraction of cations. As a consequence, the interface cannot be described in terms of a simple separation between cation- and anion-dominated regimes. Instead, the interfacial charge is continuously modulated by the balance between specific adsorption and electrostatic compensation.

The correlation between voltammetric features and the potential dependence of the SO_4_^2–^ and K^+^ signals further supports the strong coupling between adsorbate structure and interfacial ion concentration. Increases in the sulfate signal coincide with the H_UPD_ features, indicating that these peaks are not solely associated with hydrogen and OH adsorption/desorption but also involve sulfate adsorption processes. This is particularly apparent for the voltammetric peak around 0.3–0.35 V_RHE_, in agreement with radiotracer studies,^46^ highlighting the sensitivity of our technique to subtle coadsorption phenomena.

These observations again demonstrate that adsorbates have a major impact on the EDL structure, largely dictating ion accumulation in the EDL as a function of potential. In the absence of anion chemisorption, the EDL is dominated by just one type of ion (cation or anion), without much evidence of cation–anion pairing. In contrast, in the presence of specific adsorption, cation–anion coexistence does occur as a result of overscreening by the specifically adsorbed ions.

Having established the role of adsorbates and ion-specific interactions, we next examine how bulk electrolyte concentration influences interfacial charging. Figure 4(a) shows the Cl XRF intensity during cyclic voltammetry in 10 mM HClO_4_ with varying concentrations of added KClO_4_. Across all concentrations, the potential-dependent shape of the ClO_4_^–^ signal remains similar to that observed in Figure 2(a), indicating that the underlying charging mechanism is preserved. However, the absolute intensity of the interfacial anion signal above the PZFC increases systematically with bulk perchlorate concentration.

**Figure 4:**
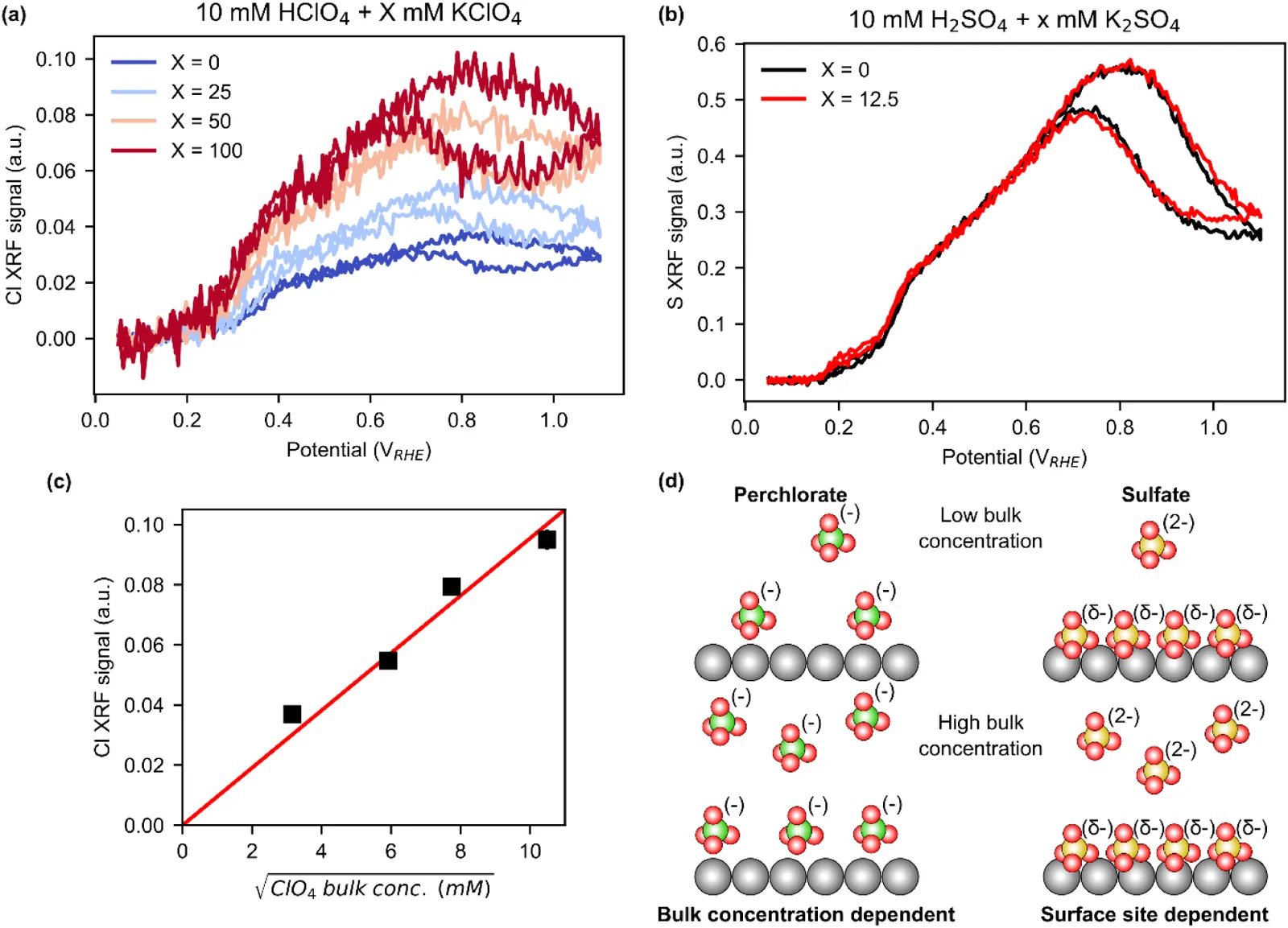
(a) Cl Kα XRF intensity as a function of potential during cyclic voltammetry measured at 10 mV/s for varying bulk concentrations of perchlorate. (b) S Kα XRF intensity as a function of potential during cyclic voltammetry measured at 10 mV/s for varying bulk concentrations of sulfate. (c) Cl Kα XRF intensity extracted at 0.8 V from (a) as a function of the square root of the bulk perchlorate concentration. (d) Schematic atomistic models of the interface at high and low bulk concentrations of perchlorate and sulfate.

This trend is quantified in Figure 4(c), which shows the Cl XRF intensity at 0.8 V as a function of the square root of the bulk perchlorate concentration. Since the EDL is fully dominated by anions at this potential (Figure 2a), the interfacial ClO_4_^–^ signal directly reflects the surface free charge density. This allows us to compare our results to the surface free charge versus concentration relationship predicted by the Grahame equation (eq 1)^47^ derived from Gouy–Chapman (GC) theory:

1 $$\sigma =\sqrt{8C_{0}\varepsilon \varepsilon_{0}k_{B}N_{A}T}\sinh\left(\frac{ze\varphi_{s}}{2k_{B}T}\right)$$

where σ is the surface free charge, *C*_0_ is the bulk ion concentration, ε is the absolute permittivity, ε_0_ is the relative permittivity, *k*_B_ is Boltzmann’s constant, *N*_*A*_ is Avogadro’s constant, *T* is the temperature, z is the valency of the ion, e is the electron charge, and φ_*s*_ is the surface potential. According to the Grahame equation, the surface free charge should follow a square root dependence on the bulk ion concentration for a fixed surface potential. The data in Figure 4(c), which is plotted on a square root scale, can be fit by a straight line passing through the origin, indicating that the behavior predicted by the Grahame equation fits the experimental data for perchlorate under these conditions (see Supporting Information Section 8, Figure S7 for linear *x*-axes).

The fact that the Grahame equation can describe the concentration-dependent experimental data for perchlorate indicates that GC theory actually partly holds under these conditions, specifically: a nonadsorbing anion (perchlorate) interacting with an electrode (platinum), which is covered by adsorbates (OH and oxide). It also indicates that charge is stored in the diffuse part of the double layer, without influence from ion packing or dielectric saturation (despite the significant bulk ion concentration), as such effects are not considered in GC theory. However, it is important to note that the sine hyperbolic potential dependence of the Grahame equation (stemming from the GC capacitance) is expected to break down at high surface free charges and high concentrations.

When considering the energy terms for double layer formation, the bulk ion concentration dependence on the surface free charge can also be rationalized in terms of thermodynamics. Increasing the electrolyte concentration decreases the entropic penalty associated with accumulating ions at the interface, as the difference in configurational entropy between ions in the bulk and in the EDL becomes smaller.^48^ As a result, the system can sustain a higher interfacial ion concentration—and thus a higher surface free charge—at the same applied potential.

In contrast to the perchlorate electrolyte, the behavior is markedly different for specifically adsorbing anions such as sulfate, which suppresses the diffuse double layer. Figure 4(b) shows the corresponding measurements in sulfate-containing electrolytes. Here, the interfacial sulfate signal exhibits little dependence on bulk concentration over the investigated range. This insensitivity indicates that the interfacial concentration is not limited by entropic considerations, but instead by the availability of adsorption sites and the strength of the sulfate–surface interaction.

In this regime, the free energy of the interface is dominated by enthalpic contributions associated with specific adsorption (Helmholtz capacitance), rather than by the entropy of ion redistribution (diffuse double layer capacitance). As a consequence, increasing the bulk sulfate concentration does not significantly alter the interfacial coverage, which is already close to saturation. This behavior further reinforces the distinction between nonadsorbing and specifically adsorbing electrolytes: while the former exhibit concentration-dependent double-layer charging governed in part by entropy, the latter are controlled primarily by surface chemistry and adsorption thermodynamics, as schematically illustrated in Figure 4(d).

Shifting focus to the negatively charged surface below ∼0.28 V_RHE_, we also studied the effect of bulk cation concentration on EDL formation. Figure 5(a) shows the K XRF intensity as a function of potential for varying bulk K^+^ concentrations. Again, we find that the general shape of the interfacial K^+^ concentration vs potential curve is maintained independent of the bulk K^+^ concentration, indicating a similar charging mechanism in all cases.

**Figure 5:**
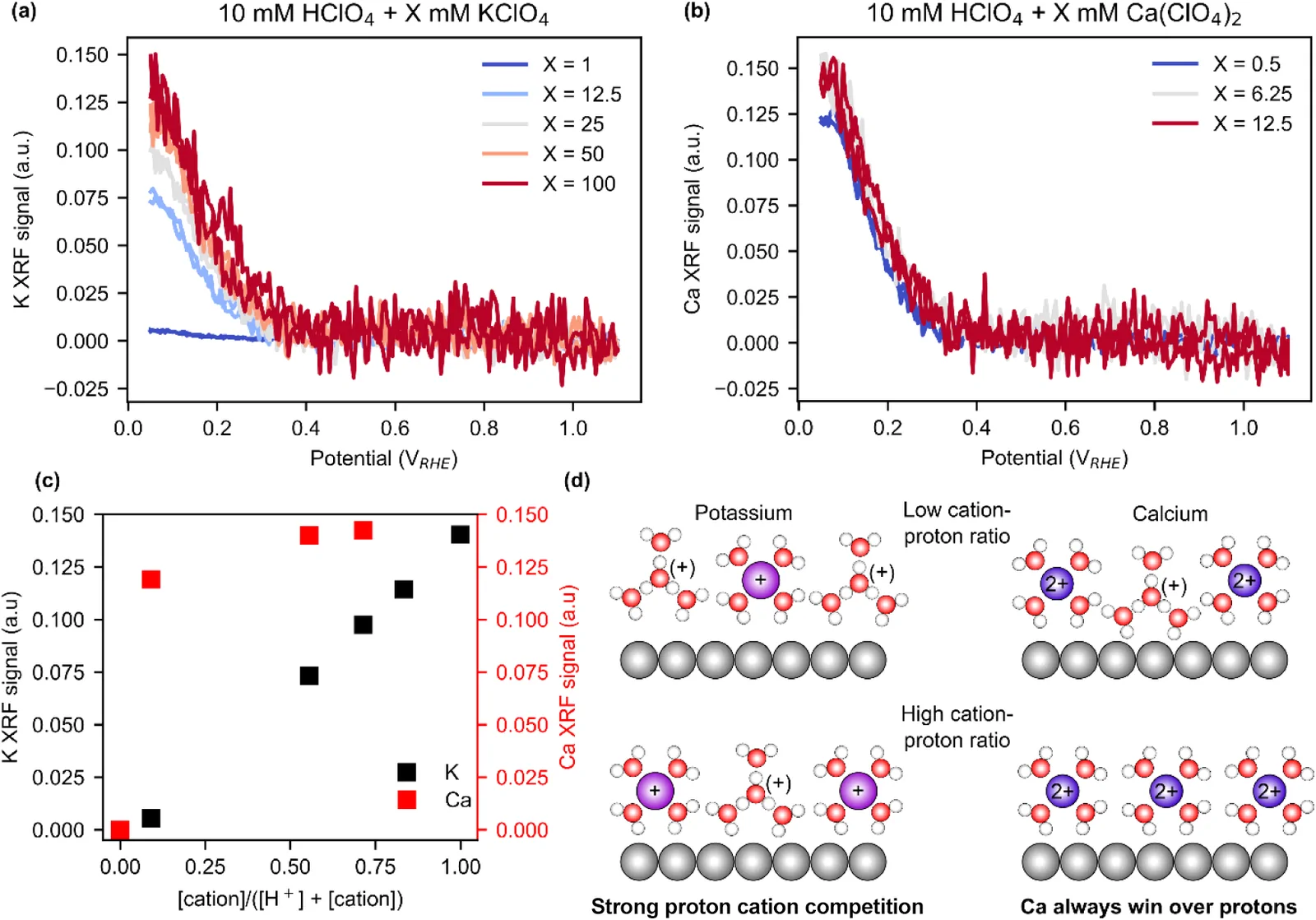
(a) K Kα XRF intensity as a function of potential for varying bulk K^+^ concentrations. (b) Ca Kα XRF intensity as a function of potential for varying bulk Ca^2+^ concentrations. (c) K and Ca Kα XRF intensities at 0.05 V as a function of the ratio between cation and total bulk ion concentration (point at 0, 0 has been added for clarity). (d) Schematic atomic models of the interface.

Similar to the perchlorate case, the magnitude of the signal depends strongly on the bulk K^+^ concentration. However, the origin of this bulk K^+^ concentration dependence is fundamentally different from that of perchlorate: it is primarily due to the competition between K^+^ and H^+^ cations. This competition is quantified in Figure 5(c), where the interfacial signal at 0.05 V is plotted as a function of the ratio between K^+^ and total bulk cation (K^+^ + H^+^) concentration (see Supporting Information Figure S8 for a linear *x*-axis). A straight line through the origin is observed, indicating that a mixed double layer of K^+^ and H^+^ forms, reflecting the bulk ion ratio of K^+^ and H^+^. This suggests that the free energy of screening the surface free charge is similar for H^+^ and K^+^. Since the cation-proton competition is thus not driven by chemically specific ion-adsorbate interactions, we anticipate that it is generalizable to other electrochemical systems, including more ideal adsorbate-free systems.

Importantly, this direct linear relationship in Figure 5c indicates that the cation-dominated EDL does not follow GC theory, as this would have resulted in a straight line on a $\left[K^{+}\right]/\sqrt{\left[K^{+}\right]+\left[H^{+}\right]}$ axis (see Supporting Information Figure S9 for a plot with this axis). In fact, after correcting for the H^+^/K^+^ competition, it can be observed that the surface free charge only marginally depends on the total cation concentration in the bulk electrolyte, as shown in the Supporting Information Figure S10. This suggests that the EDL containing cations (below PZFC) does not store charge in the diffuse double layer like in the ClO_4_^–^ dominated EDL, but follows more Helmholtz capacitance-like behavior.

For the divalent Ca^2+^ cation, we find even stronger deviation from Gouy–Chapman behavior. Figure 5(b) shows the Ca XRF signal for varying Ca^2+^ concentrations. In contrast to the K^+^ case, the Ca^2+^ signal shows almost no dependence on the bulk Ca^2+^ concentration. This indicates that Ca^2+^ is strongly favored over H^+^, meaning that Ca^2+^ wins the ion competition, and that we are far from Gouy–Chapman behavior. The observed saturation could point toward local dielectric saturation or ion crowding, caused by the stronger electrostatic interaction of divalent ions with the negatively charged surface or by the fact that the charge is stored in the inner layer and not in the diffuse part of the double layer.

## Conclusion

In this work, we provide a direct, element-specific view of EDL formation at the Pt-electrolyte interface in acidic media by combining cyclic voltammetry with *in situ* resonant XRF. Our results demonstrate that interfacial charging cannot be described solely in terms of classical electrostatic models, but is shaped by the coupled interplay of adsorbates, bulk ion concentration, and ion competition.

Adsorbates define the effective PZFC, inducing nonmonotonic charging behavior that strongly deviates from classical theories. For a fixed adsorbate structure, however, the EDL can still behave close to ideal depending on the type of ions present. For the perchlorate ion, we find that the Gouy–Chapman model accurately captures double layer charging as a function of bulk ion concentration, indicating that the charge is stored in the diffuse EDL (GC capacitance). In contrast, cations such as K^+^ and Ca^2+^ show Helmholtz capacitance behavior, with little dependence of the interfacial cation concentration on the (total) bulk cation concentration. Instead, the composition of the double layer is determined by the competition between protons and other cations, with a strong dependence on ion valency. The understanding of these fundamental differences in the behaviors commonly used ions provides new handles for moving beyond classical EDL models. Taking the quantitative interfacial ion accumulation data provided here as a benchmark, we anticipate that our work will thus spark new developments in the EDL modeling, accounting for adsorbates inducing nonmonotonic charging, overscreening, and cation-proton competition in mixed EDLs.

## Methods

The *in situ* resonant XRF measurements were carried out at the LUCIA beamline at the SOLEIL synchrotron. The tender X-ray beamline provides monochromatized photon beams with energies from 0.8 to 8 keV, allowing measurements of SO_4_^2–^, ClO_4_^–^, K^+^, and Ca^2+^ ions at the K-edges of S (∼2472 eV), Cl (∼2822 eV), K (∼3608 eV), and Ca (∼4038 eV), (for full XAS spectra, see Supporting Information Section 10, Figures S11–S14). The fluorescence X-rays were detected with a silicon drift detector (SDD). A peristaltic pump was used to deliver the electrolyte, which was not recirculated to avoid the accumulation of beam damage in the electrolyte (e.g., ClO_4_^–^ decomposition to form Cl^–^). Cl K-edge spectra before and after time-resolved measurements during CVs are shown in the Supporting Information Section 11, Figure S15, indicating the absence of beam induced damage since the intensity and shape of the spectrum is preserved. At high X-ray doses this is not the case.

A custom-designed spectro-electrochemical flow cell with a three-electrode configuration that can accommodate a 1 × 1 cm^2^ SiN_*x*_ chip was used. A Pt wire was used as the counter electrode in combination with a miniature leakless Ag/AgCl reference electrode (Innovative Instruments). All potentials were converted to the reversible hydrogen electrode (RHE) scale using *E*_RHE_ = *E*_Ag/AgCl_ + 0.197 V + 0.059 × pH.

Time resolved XRF signals were measured using an X-ray energy corresponding to the white line energy of each respective element, S: 2482 eV, Cl: 2834.5 eV, K: 3616 eV, Ca: 4050.5 eV. The XRF signals were extracted by integrating the Kα peaks in the XRF spectra recorded by the SDD. This was done by integrating the signal in the following energy ranges for each respective element, S: 2262–2411 eV, Cl: 2486–2685 eV, K: 3084–3432 eV, Ca: 3482–3830 eV). The reproducibility of the time-resolved signals is highlighted in Supporting Information, Section 12.

Electrolytes were prepared using ultrapure water and HClO_4_ (Carl ROTH, ROTIPURAN Ultra 70%), H_2_SO_4_ (Supelco, 96%, Suprapur), KOH (Fluka, Honeywell, TraceSELECT, ≥99.995% metals basis), and Ca­(OH)_2_ (Sigma-Aldrich 99.995% trace metals basis). The KOH was further purified by using nickel hydroxide particles as metal ion scavengers, as described in refs 49–51 , and the consequent removal of the particles by centrifugation and nanofiltration with a 0.22 μm poly­(ether sulfone) filter. The acidic electrolytes were prepared by recombining high-purity bases and acids to achieve the desired alkali or alkali earth cation concentration and pH. This method was selected as KClO_4_ and Ca­(ClO_4_)_2_ are not commercially available in the same high purities as the hydroxide salts. The electrolyte compositions are stated in the main text.

All cyclic voltammograms are measured at 10 mV/s in the potential range of 0.05–1.1 V_RHE_. A biologic VSP-300 potentiostat was used for all electrochemical experiments. The potentials were *iR*-corrected by 85% in the potentiostat software, with the solution resistance determined from the high-frequency region of the electrochemical impedance spectra. The last 15% were corrected postanalysis.

All glassware and the flow cell were cleaned according to the following procedure. Items were soaked overnight in 0.5 M H_2_SO_4_ (Sigma-Aldrich, 95.0–97.0%) containing 1 g L^–1^ KMnO_4_ (Emsure, ≥99.0%) to remove organic contaminants. They were then rinsed thoroughly with ultrapure water (Milli-Q, resistivity ≥ 18.2 MΩ·cm) and soaked for 10 min in freshly prepared diluted piranha solution (H_2_O_2_, Merck, 35%; H_2_SO_4_, Sigma-Aldrich, 95.0–97.0%) to remove residual manganese oxides. After the piranha treatment, the items were rinsed with ultrapure water and boiled three times. For the EQCM flow cell, both piranha solution and ultrapure water were circulated through the cell and tubing to ensure complete cleaning.

The mesoporous Pt film was deposited onto a 20 nm Au/SiN_*x*_ chip (Silson, 100 nm 1 × 1 mm^2^ window), via a soft templating process.^52^ Prior to deposition, the Au-coated SiN_*x*_ chips were cleaned by soaking in a dilute piranha solution for 1 min and afterward rinsed in ultrapure water. Deposition was carried out in a three electrode electrochemical cell with a Pt wire as counter electrode and a miniature leakless Ag/AgCl reference electrode (Innovative Instruments) using an electrolyte solution containing Brij 58 (0.2455 g), which was dissolved in 20 mL ultrapure water, followed by the addition of 2.46 mL of 8 wt % hexachloroplatinic acid (HCPA) solution. Deposition was carried out at around 0 V vs Ag/AgCl until a charge of 1500 mC/cm^2^ had passed through the cell, resulting in a film thickness of around 750 nm. After the deposition, the sample was rinsed and soaked in ethanol and ultrapure water to remove any remaining surfactant within the film.

## Supplementary Material



## Notes

Structural characterization (SEM, SAXS), discussion of the absence of double-layer overlap, mass transfer and potential gradients within the porous film, discussion of the absence of self-absorption, EQCM data, full XAS spectra, full time-dependent XRF signals, and XRF data treatment (PDF)
