# Supplementary material for: Nonmonotonic Charging, Overscreening, and Ion Competition in the Electric Double Layer of Platinum Quantified by X‑ray Spectroscopy
Source: J Am Chem Soc. 2026 Sep 26;148(39):42623–31. doi: 10.1021/jacs.6c14786 (PMC13651505; doi:10.1021/jacs.6c14786)
Supplement: Supplementary file 1 [file ja6c14786_si_001.pdf]

Supporting Information for:

Non-monotonic Charging, Over-screening, and Ion Competition in the Electric Double Layer of Platinum Quantified by X-Ray Spectroscopy

Alfred Larsson[1], Dogukan Yilmaz[1], Jan Sebastian Dominic Rodriguez[1,2], Tarik Zahzah[1], Pieter de Haij[1], Jordy J.J. Eggebeen[1], Karen van den Akker[1], Damien Roy[3], Solenn Reguer[3], Marc T.M. Koper[1], Rik Mom[1*]*

[1]Leiden Institute of Chemistry, Leiden University, PO Box 9502, 2300 RA Leiden, The Netherlands

[2]Department of Physics, Tamkang University, New Taipei City, 251301, Taiwan

[3]Synchrotron Soleil, L'Orme des Merisiers, départementale 128, 91190 Saint-Aubin, France

**Corresponding authors: email: alfred.larsson@fysik.lu.se, r.mom@lic.leidenuniv.nl*

1: Structural characterization

Cross-sectional SEM analysis reveals a mesoporous film with homogeneous coverage and thickness, as shown in Figure S1. The relationship between measured film thickness and the deposition charge can be exploited to calculate the porosity of the film using:

$$Porosity = 100\% \times \frac{t_{measured} - t_{calculated}}{t_{measured}} \qquad \text{(eq S1)}$$

In which $t_{measured}$ is the measured film thickness and $t_{calculated}$ is the film thickness expected for a non-porous Pt film based on the deposition charge and known density of metallic Pt. Based on this analysis, we find a porosity of around 50% for our films.
By combining the porosity with the roughness factor measured using the H_{upd} features in the cyclic voltammogram, we can estimate the average pore size (assuming cylindrical pores):

$$d_{pore} = 4 \times \frac{V_{Pore}}{A_{Pore}} = \frac{4 \times t_{measured} \times Porosity}{RF} \qquad \text{(eq S2)}$$

With d_{pore}, V_{pore} and A_{pore} the pore diameter, volume and area, respectively, and RF the roughness factor. From this analysis, a pore diameter of ~10 nm is found. Note that this method of pore diameter measurement is prone to overestimation, as voids in the film may contribute significantly to the porosity.

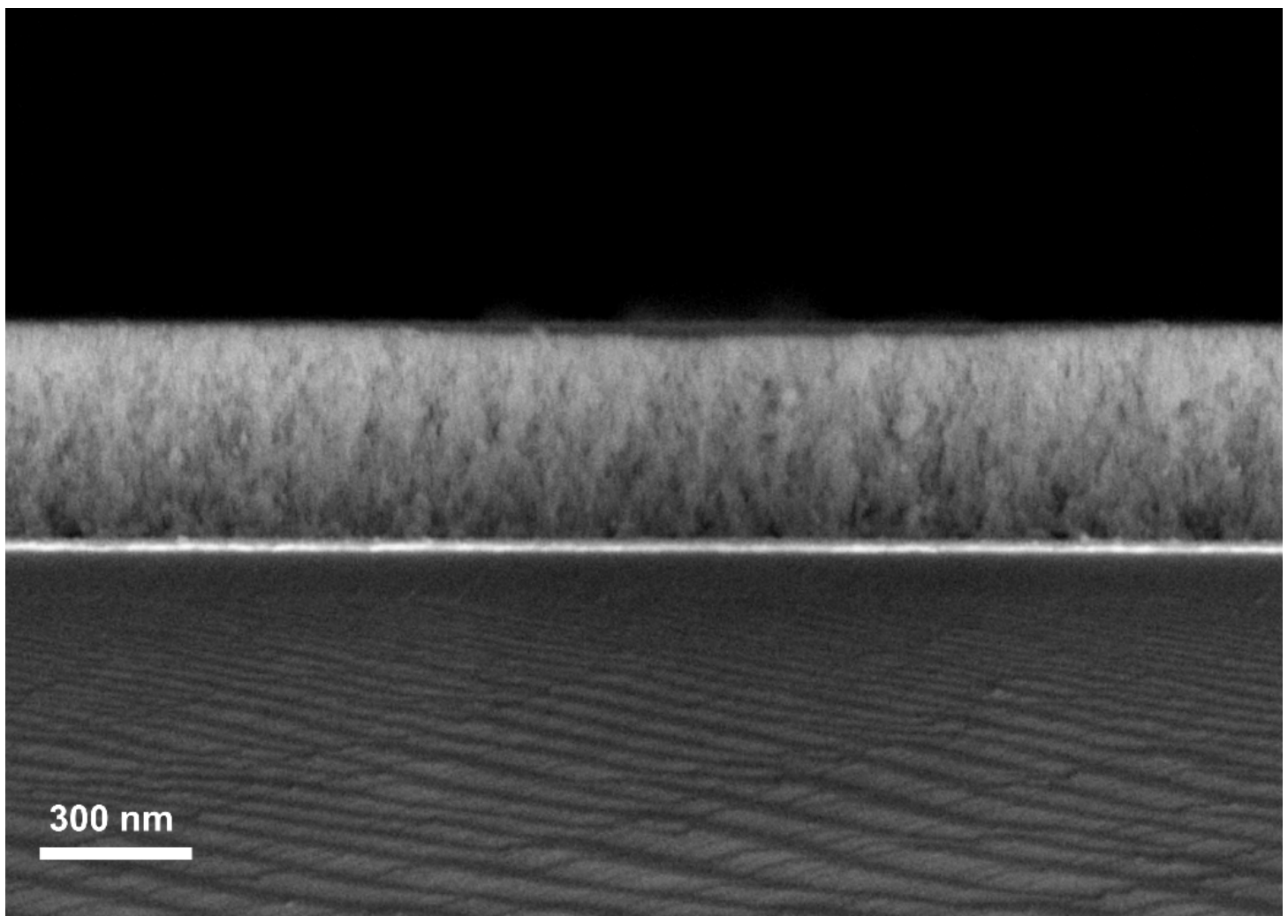

Figure S1: SEM cross-sectional image of a mesoporous sample grown using 1000 mC/cm^2 (500 nm thickness)

Further analysis was therefore performed using small-angle X-ray scattering (SAXS). Using SAXS, we characterized the pore morphology and local distances in the porous film, as shown in Figure S2. A clear peak is observed at Q=0.82 nm^{-1}, corresponding to a real-space periodic spacing of 7.7 nm, that can be assigned as the pore-to-pore distance. This pore-to-pore distance is slightly smaller than the rough pore size estimation from SEM, suggesting that the SEM pore size was indeed somewhat overestimating the real pore diameter. Rather, the SAXS data is consistent with a pore diameter close to the micellar size of the employed Brij58 soft template, which is ~7 nm.

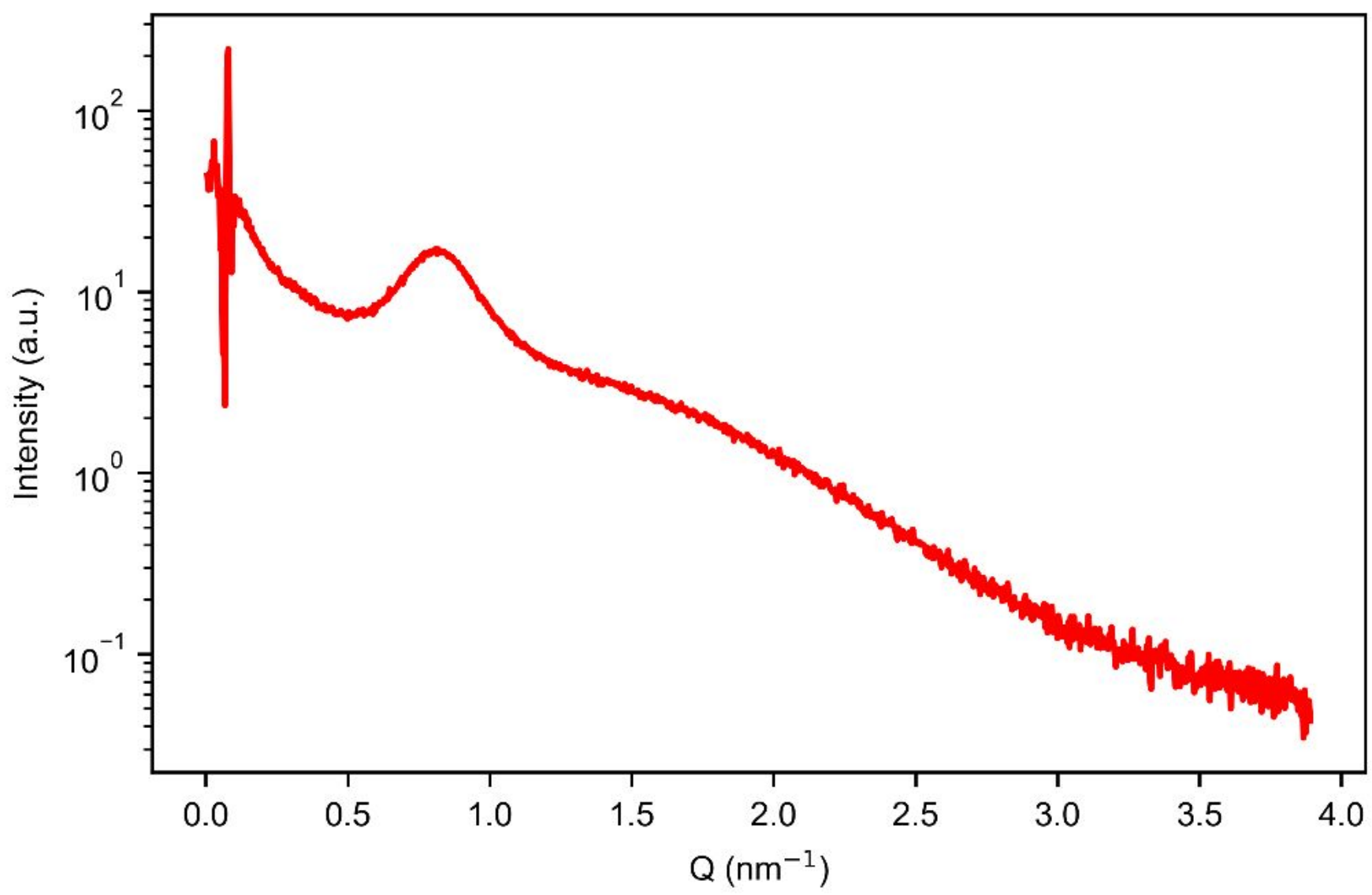

Figure S2: SAXS data for a mesoporous sample grown using 1000 mC/cm^2 (500 nm thickness)

2: Absence of double-layer overlap

Using the pore diameter of ~7 nm identified above, we can assess the possibility of double layer overlap inside the pores. At an ionic strength of 35 mM (most commonly used in this manuscript), the Debye length is 1.6 nm, which is significantly lower than the pore radius (3.5

nm), indicating that the charge is screened without double-layer overlap. We therefore do not attribute any of the observations in the manuscript to double-layer overlap.
To confirm this assessment experimentally, we analyze CVs in electrolytes with different ionic strengths. If double-layer overlap occurs, (part of) the electrode surface will be less responsive to the potential, leading to lower currents in the CV. When the ionic strength is varied, the extent of this current lowering should change as well. However, when comparing the CV measured at 10 mM $HClO_4$ with additions of 1 or 100 mM $KClO_4$, no significant difference in the current magnitude is observed, indicating no double-layer overlap, as shown in Figure S3.

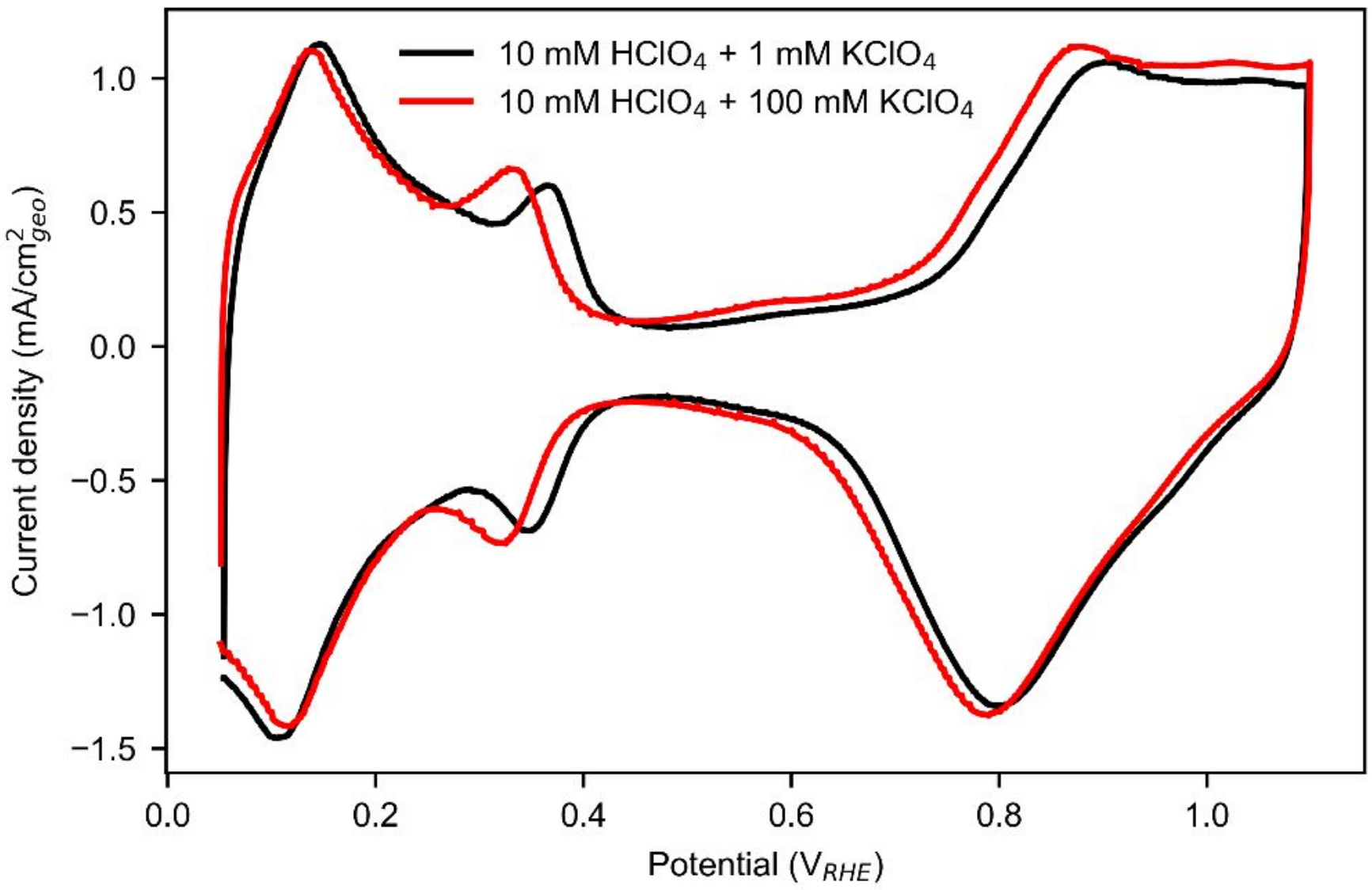

Figure S3: CV in 10 mM $HClO_4$ with additions of 1 or 100 mM $KClO_4$ for the mesoporous Pt electrode.

3: Absence of mass transfer issues

Diffusion of ions within the mesoporous Pt layer is fast enough to avoid mass-transfer limitations during dynamic (cyclic voltammetry) measurements. The diffusion time (t) can be approximated as shown in equation eq S3,

$$t = \frac{x^2}{2D} \qquad \text{(eq S3)}$$

where x is the distance, and D is the diffusion coefficient (=1.96e-5 for K^+ in water). For a diffusion length of 750 nm (equal to the thickness of the porous layer) the diffusion time is 0.14 ms. During a CV with a scan rate of 10 mV/s, the diffusion time is fast compared to the scan rate. We also see that the shape of the potential-dependent ion signals is reversible except for the hysteresis caused by oxide formation (where the CV also shows hysteresis). Furthermore, the oxidation/reduction features in the H_{upd} peaks are symmetric, also experimentally confirming that mass transfer effects are minimal.
Note that in this manuscript, we don't deal with catalytic reactions that deplete any reactant. We only study the reorganization of ions and adsorbates in response to the electrode potential.

4: Absence of potential gradients within the porous structure

Along the same lines, we can also exclude potential gradients within the porous structure. Since the conductivity of Pt is high, there is no significant potential drop across the porous layer. Furthermore, due to the absence of mass transfer limitations (and the absence of electrocatalytic reactions), potential gradients also don't build up within the in-pore electrolyte. Hence, all

pores experience the same electrode potential, and there is no potential gradient within the porous structure from top to bottom.

5: Absence of self-absorption

The tender X-rays are highly attenuated in the mesoporous Pt electrode, as shown in Figure S4. As can be seen, neither the K-edge of Cl nor that of K contributes significantly to the absorption cross section in the porous electrode, even when a very high ion concentration of 1 M is assumed within the pores to account for EDL formation: such a contribution would be visible as a jump in the attenuation length at the K and Cl K-edges, as can be seen in the case of bulk electrolyte without mesoporous film. Since the ions do not contribute to the attenuation length, we can be sure that the same volume is probed independent of the (interfacial) ion concentration. The consequence of this is that there are no self-absorption effects in our analysis, so that the XRF and XAS signals scale linearly with the ion concentration.

The attenuation length remains below 1 μm across all X-ray energies used in the manuscript, ensuring that the majority of the signal originates from ions within the pores of the mesoporous electrode (750 nm thick).

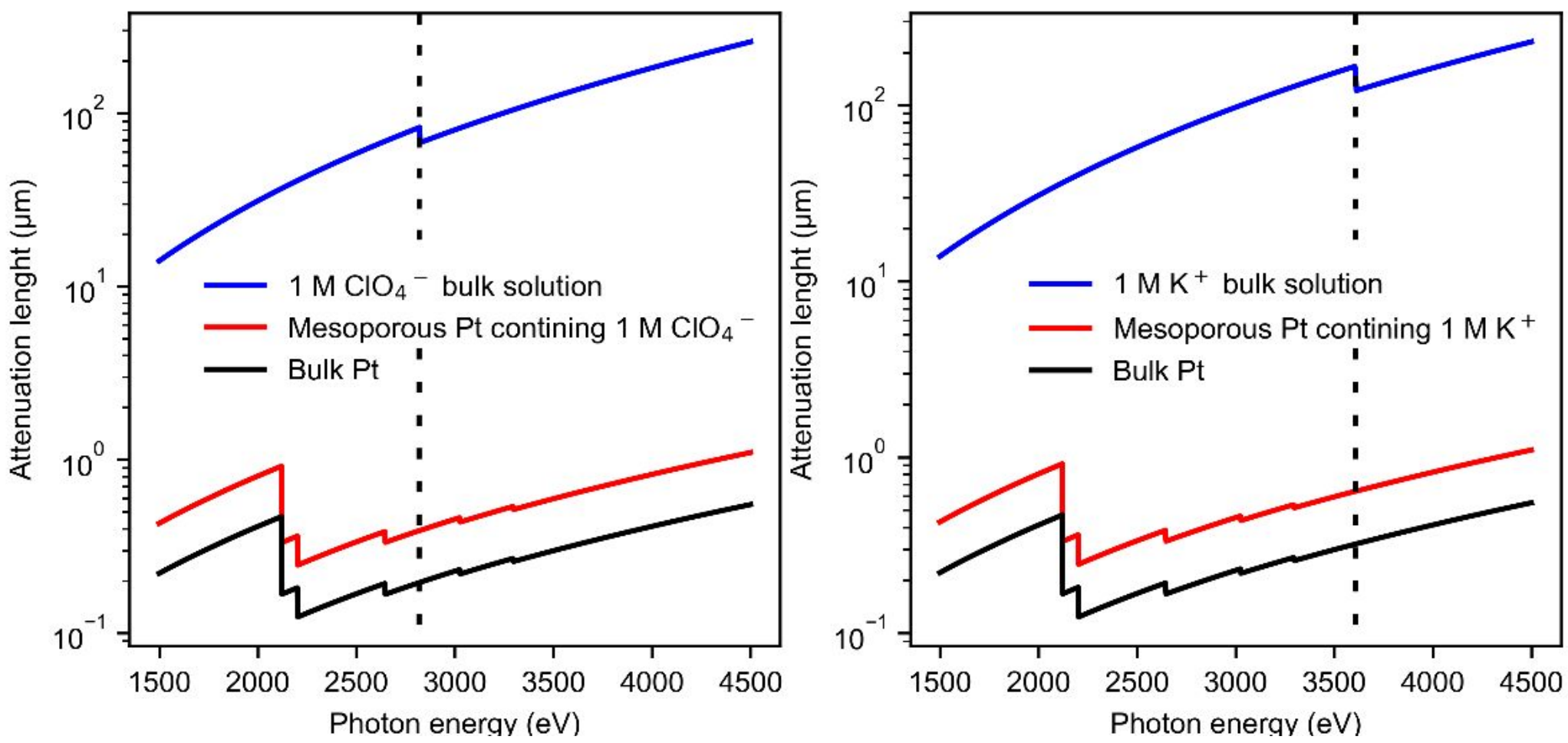

Figure S4: Attenuation length as a function of photon energy for bulk ion solutions, mesoporous electrodes with water and ions, and bulk Pt.

6: EQCM

EQCM was used to quantify ionic mass contributions to the EDL. Using a heavy ion, such as Cs^+ and comparison with a "blank" electrolyte of pure $HClO_4$ or H_2SO_4, the contribution of cations to the EDL can be visualized. The frequency decrease is proportional to the mass increase of the porous electrode, where the ions in the EDL contribute to the mass. Hence, whenever the frequency is lower in the Cs^+ containing electrolyte compared to the blank electrolyte, this means that Cs^+ was part of the EDL. Due to drift in the EQCM frequency over time, it was necessary to define the 0 of the frequency scale for every measurement. This means that the curves with and without Cs^+ had to be aligned with respect to eachother. Based on the XRF data in the main text, we set the with and without Cs^+ EQCM signals as equal at 1.1 V_{RHE}, as the data indicate little to no cations in the EDL at this potential. This approach is validated by the good curve overlap observed in Fig. S5 at anion-dominated potential ranges. Electrolytes were made using the recombination of CsOH and $HClO_4$ or H_2SO_4, and the CsOH was purified as described in the main manuscript.

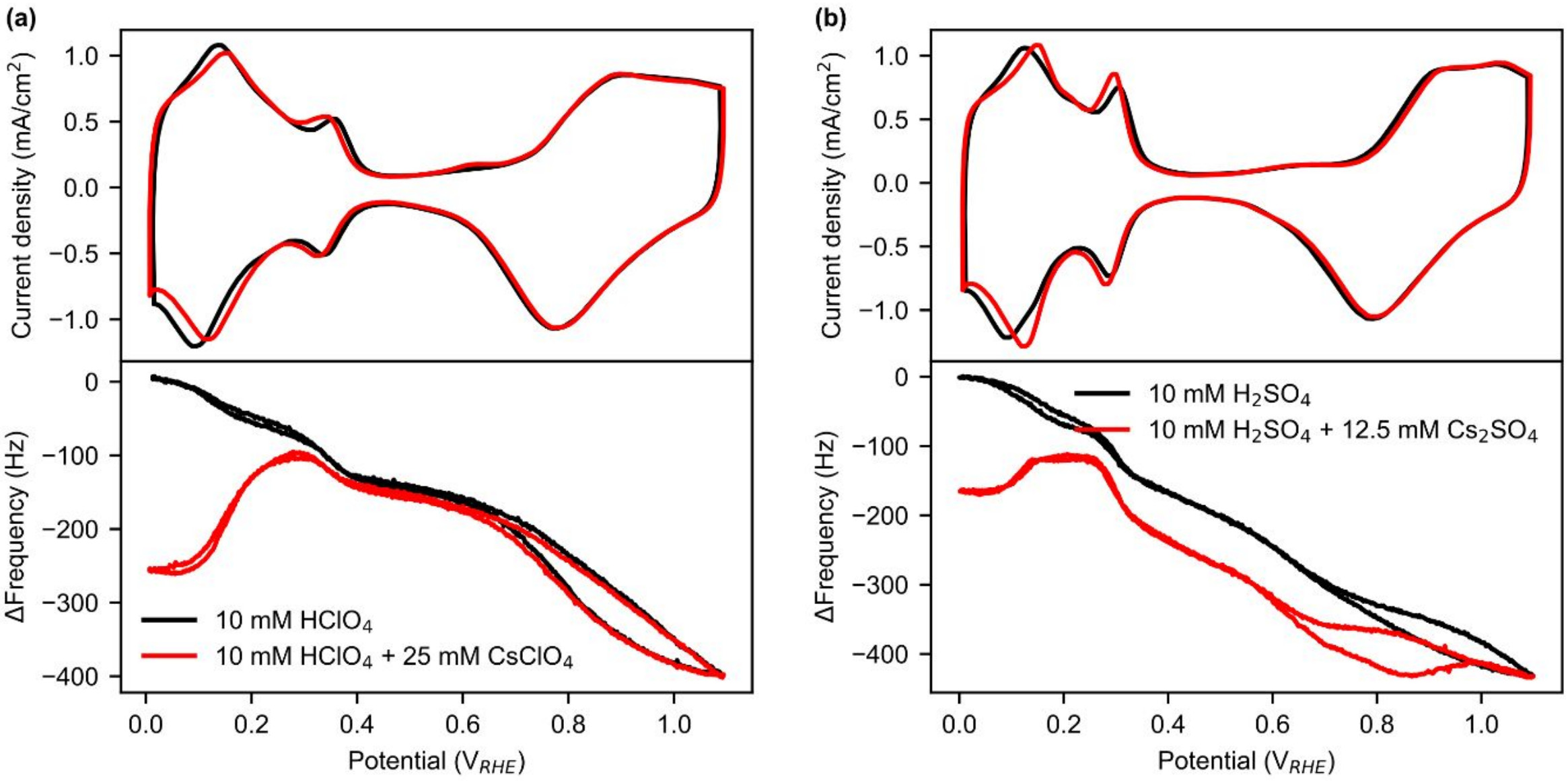

Figure S5: CV and EQCM for mesoporous Pt electrodes with and without the addition of Cs^+ ions in (a) perchloric acid and (b) sulfuric acid.

7: pH-independent PZC

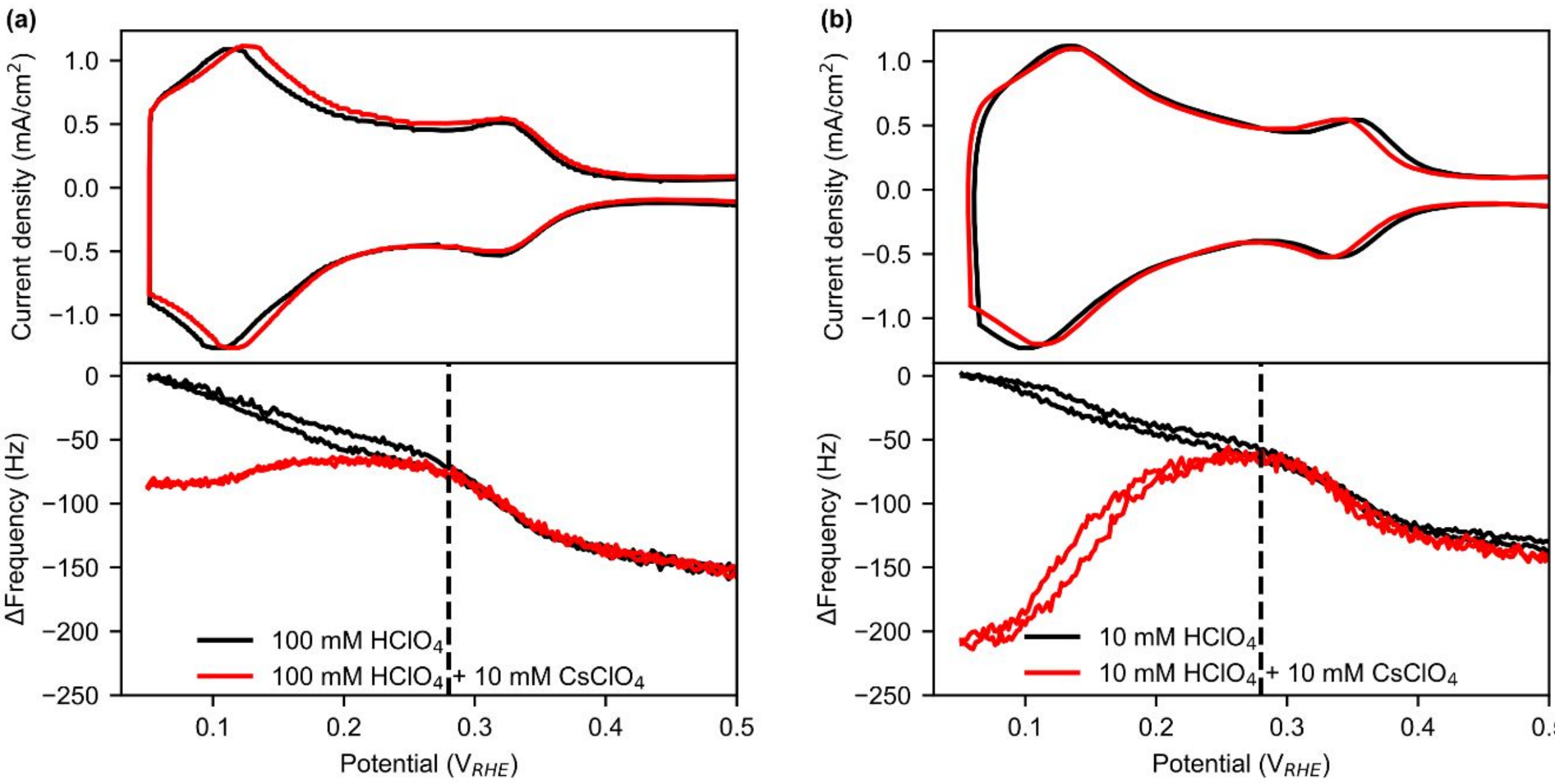

Figure S6: CV and EQCM for mesoporous Pt electrodes with and without the addition of Cs^+ ions in (a) pH 1 perchloric acid and (b) pH 2 perchloric acid. Note that the potential where Cs^+ starts being attracted, marked by dotted lines, is independent of pH.

8: $HClO_4$ signal vs concentration

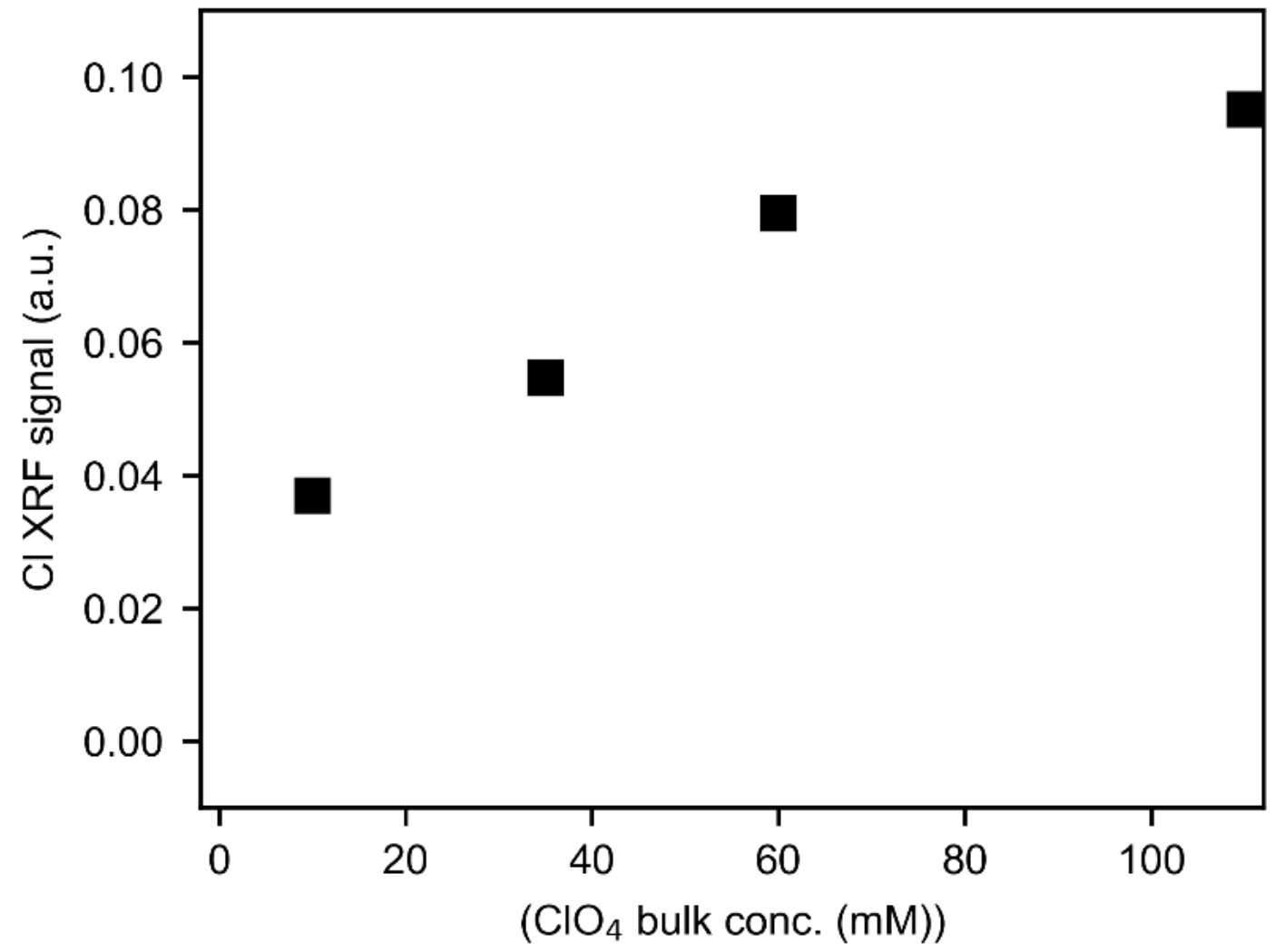

Figure S7: Perchlorate signal vs bulk perchlorate concentration on a linear scale.

9: Cation proton competition

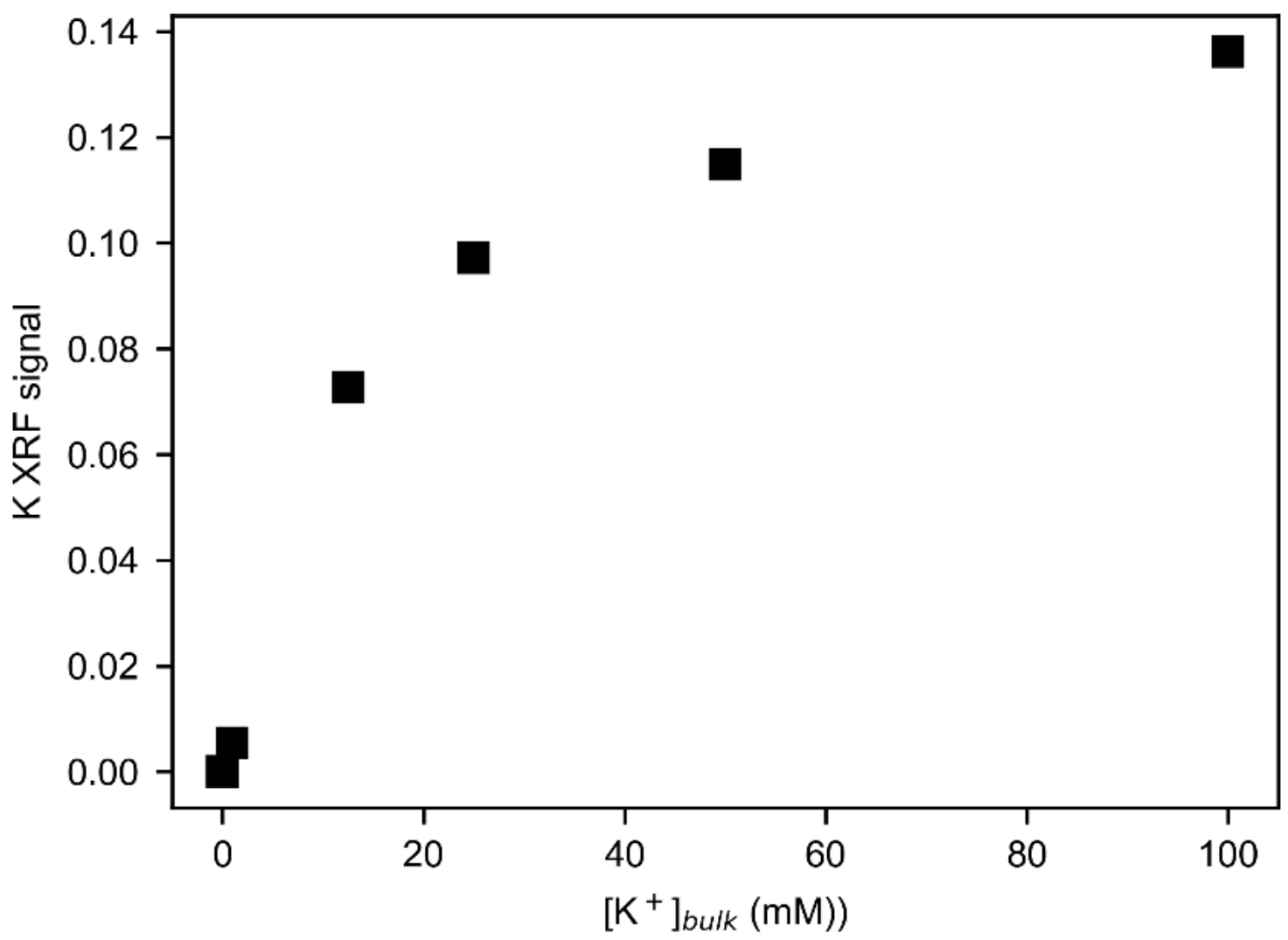

Figure S8: Potassium signal vs the bulk potassium concentration on a linear scale.

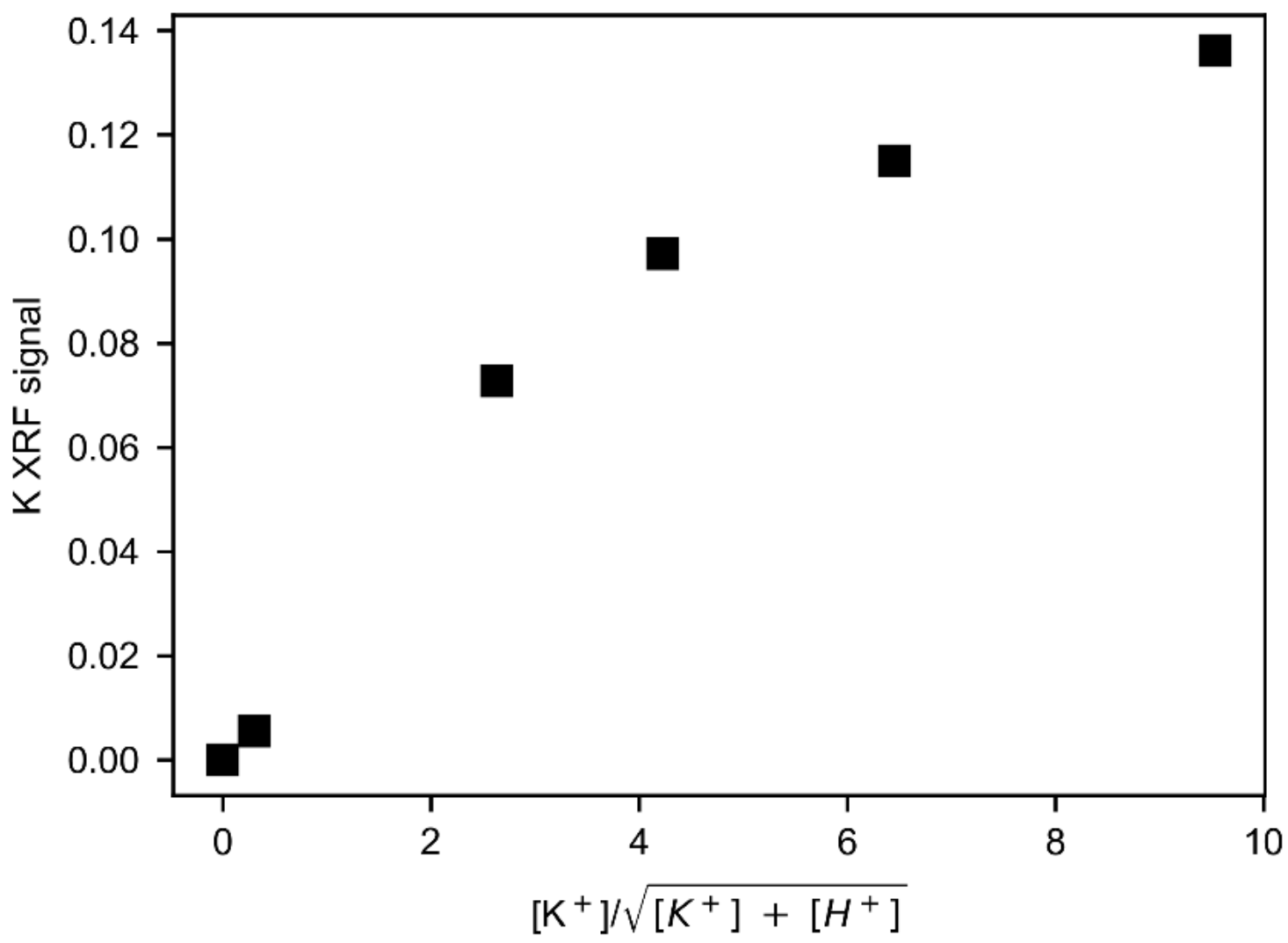

Figure S9: Potassium signal vs the square root of the total ion concentration multiplied by the potassium concentration, which would be representative of the GC model and statistical H^+/K^+ competition.

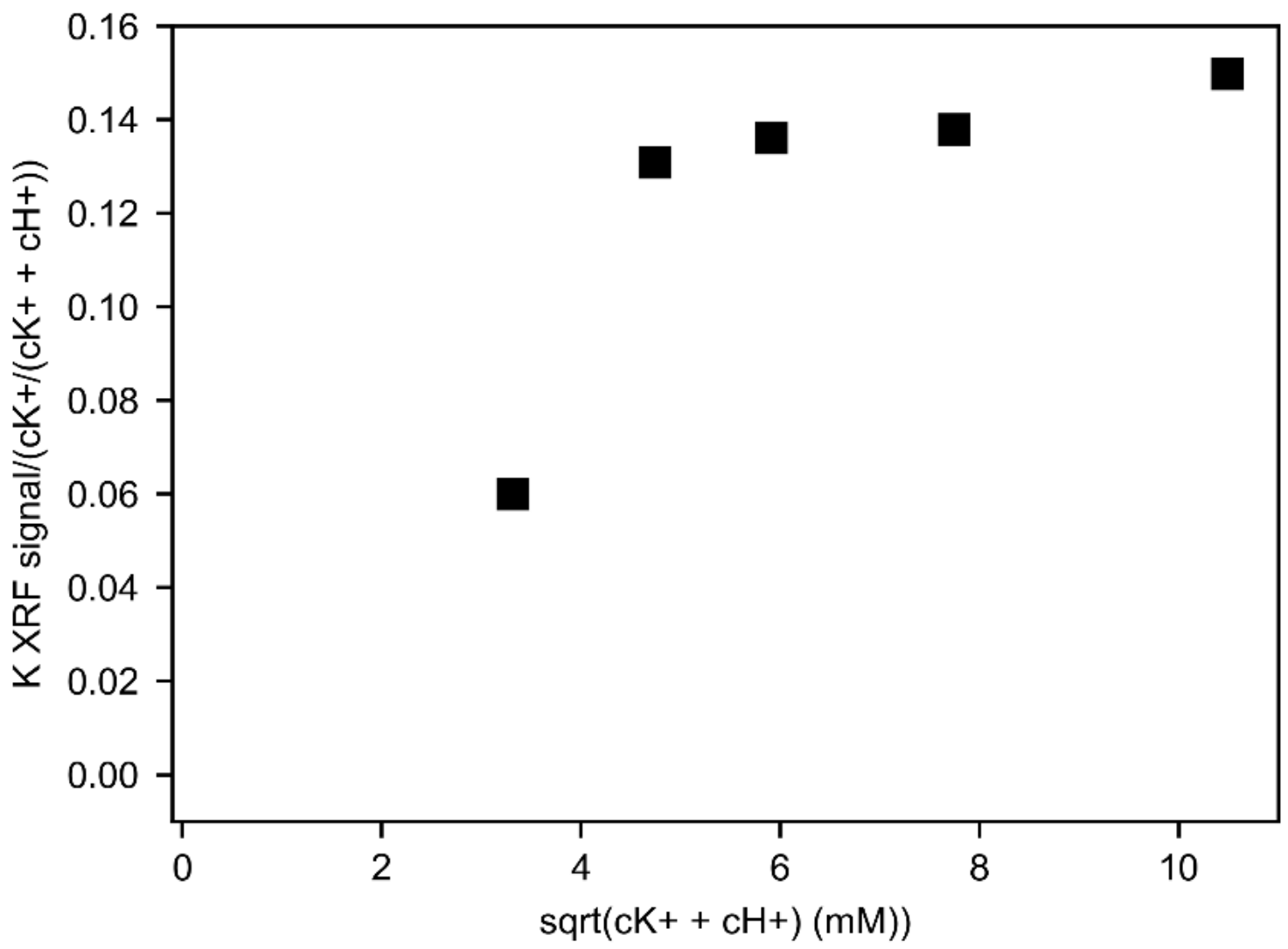

Figure S10: Potassium signal corrected for ion competition vs the square root of the total ion concentration.

10: X-ray Absorption spectra

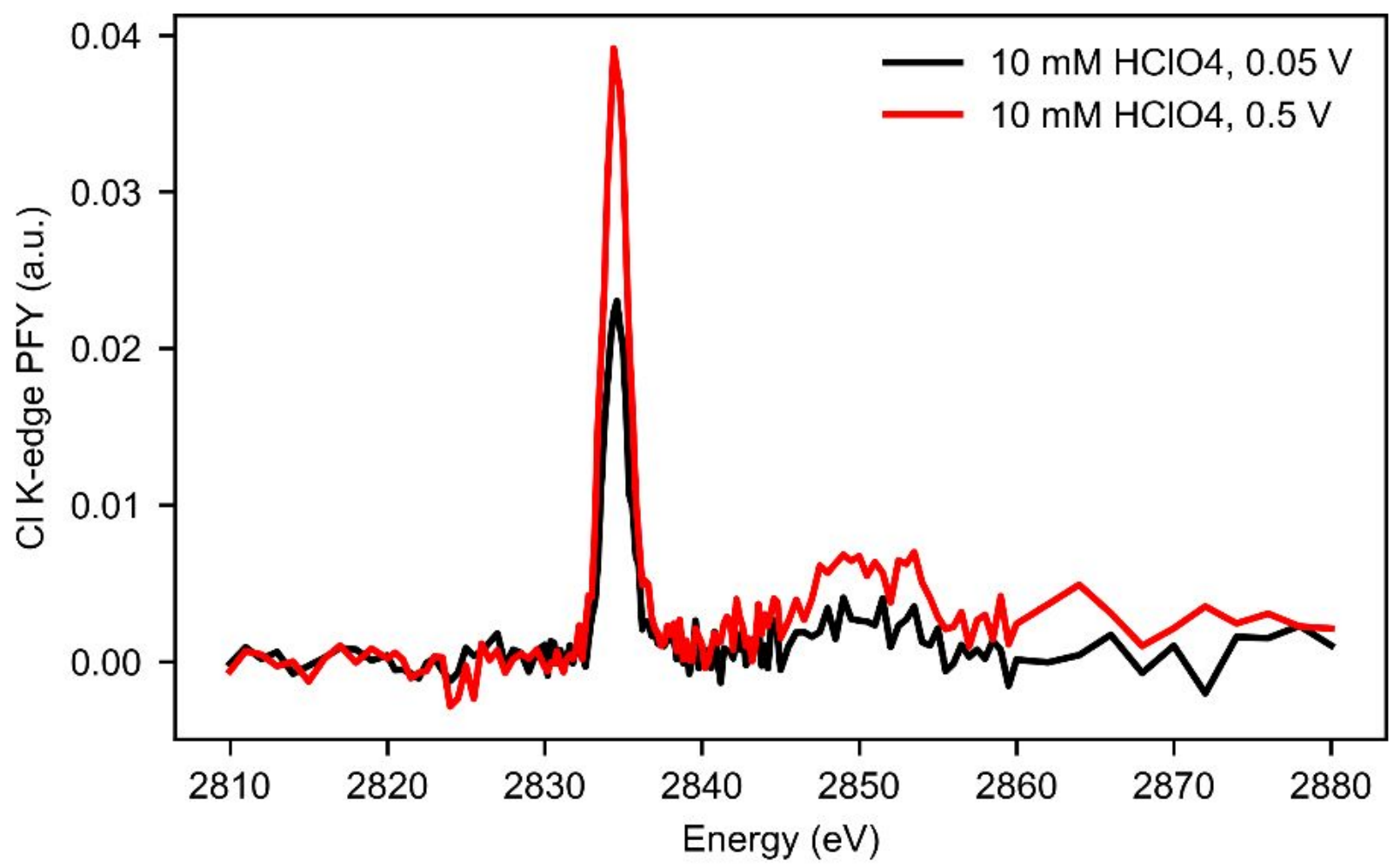

Figure S11: Cl K-edge XAS spectra at different potentials.

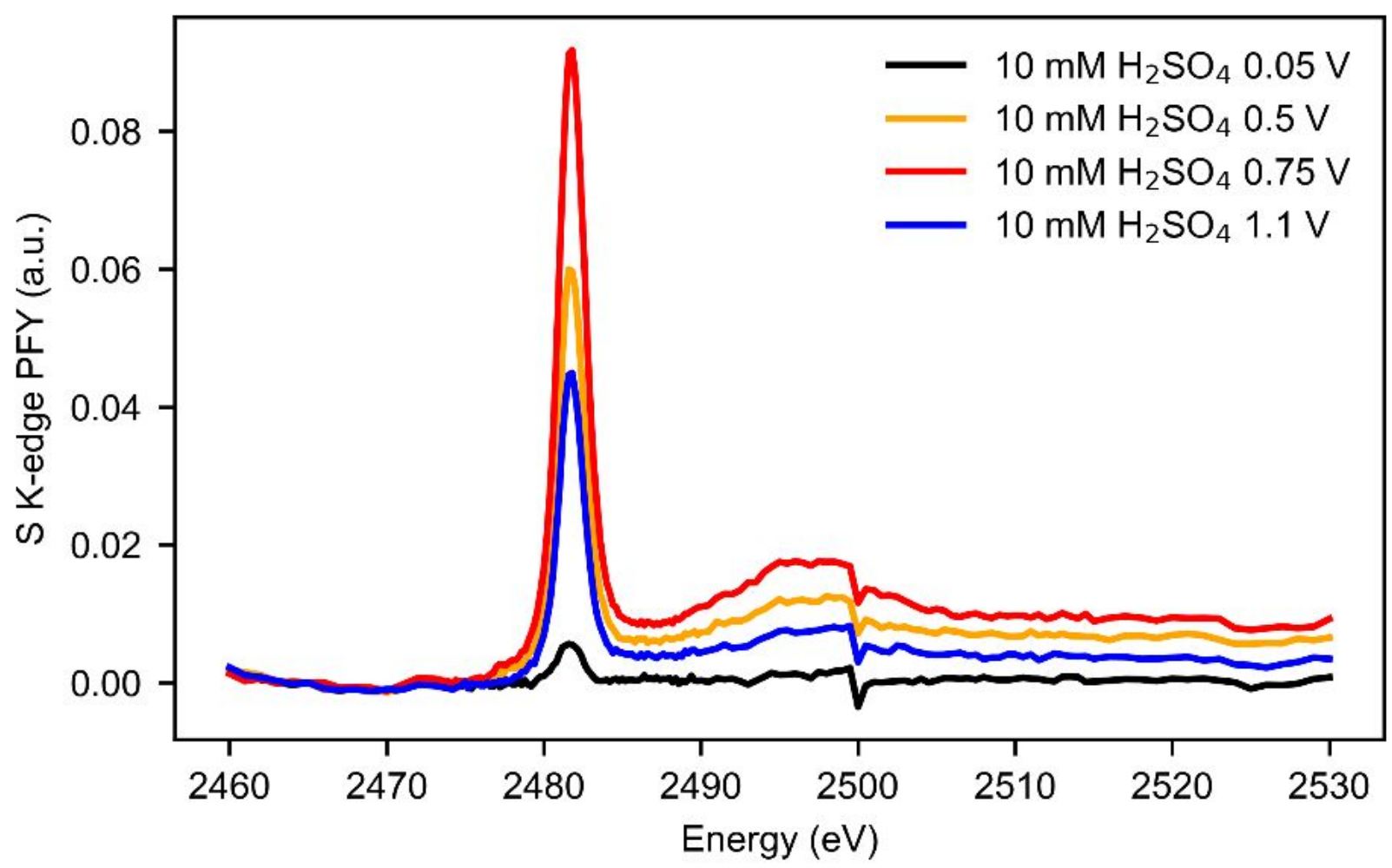

Figure S12: S K-edge XAS spectra at different potentials.

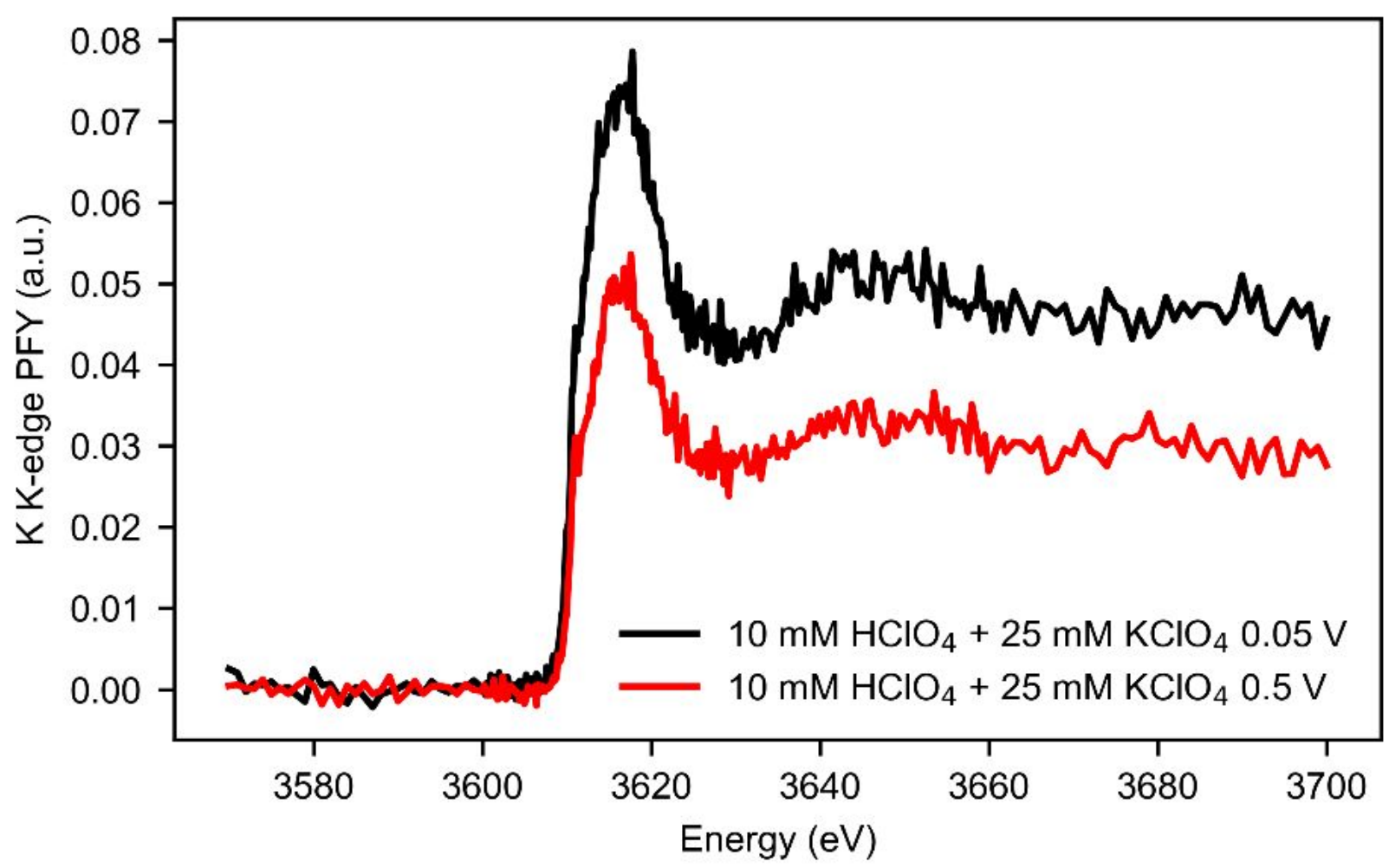

Figure S13: K K-edge XAS spectra at different potentials.

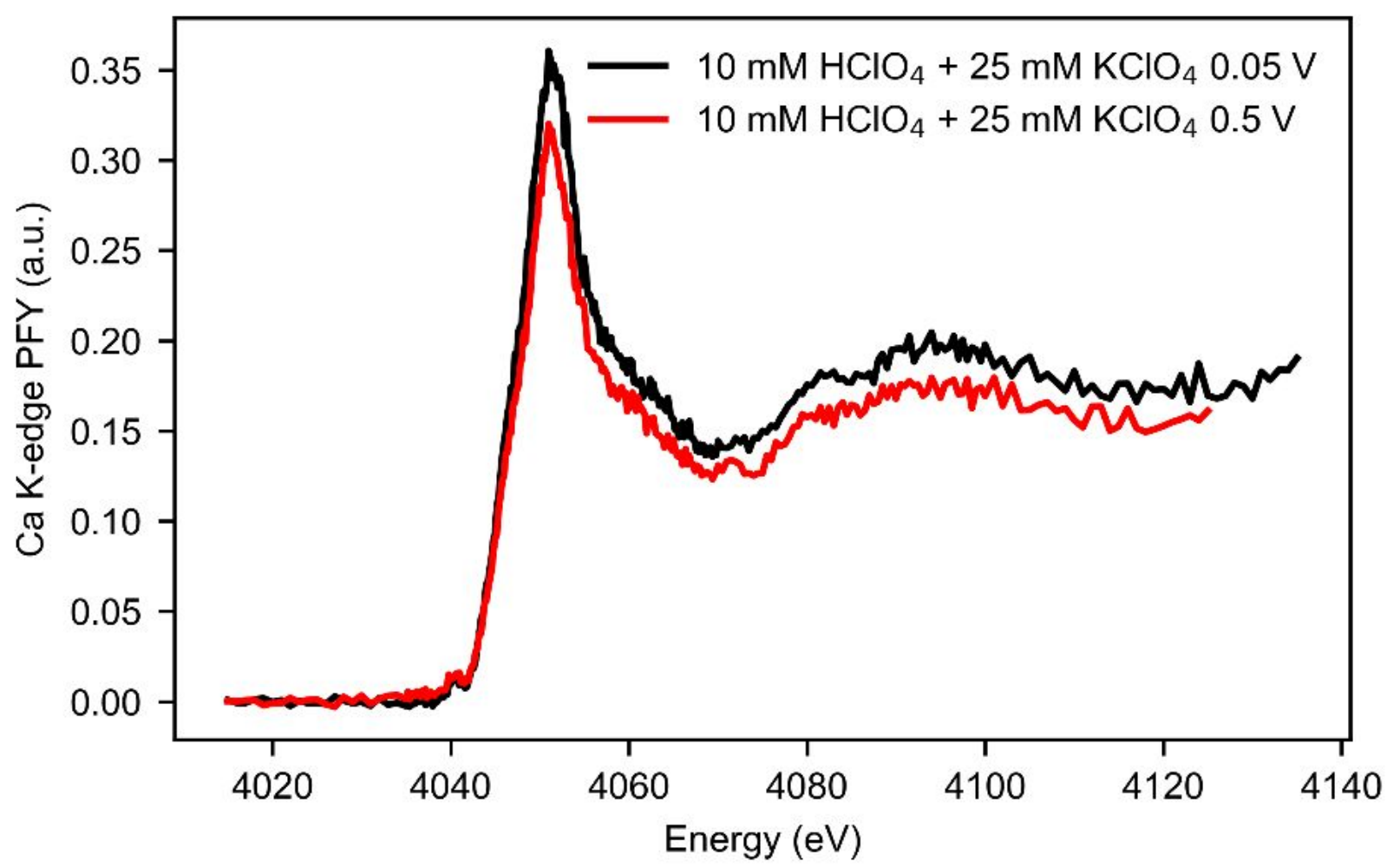

Figure S14: Ca K-edge XAS spectra at different potentials.

11: Absence of beam-induced damage

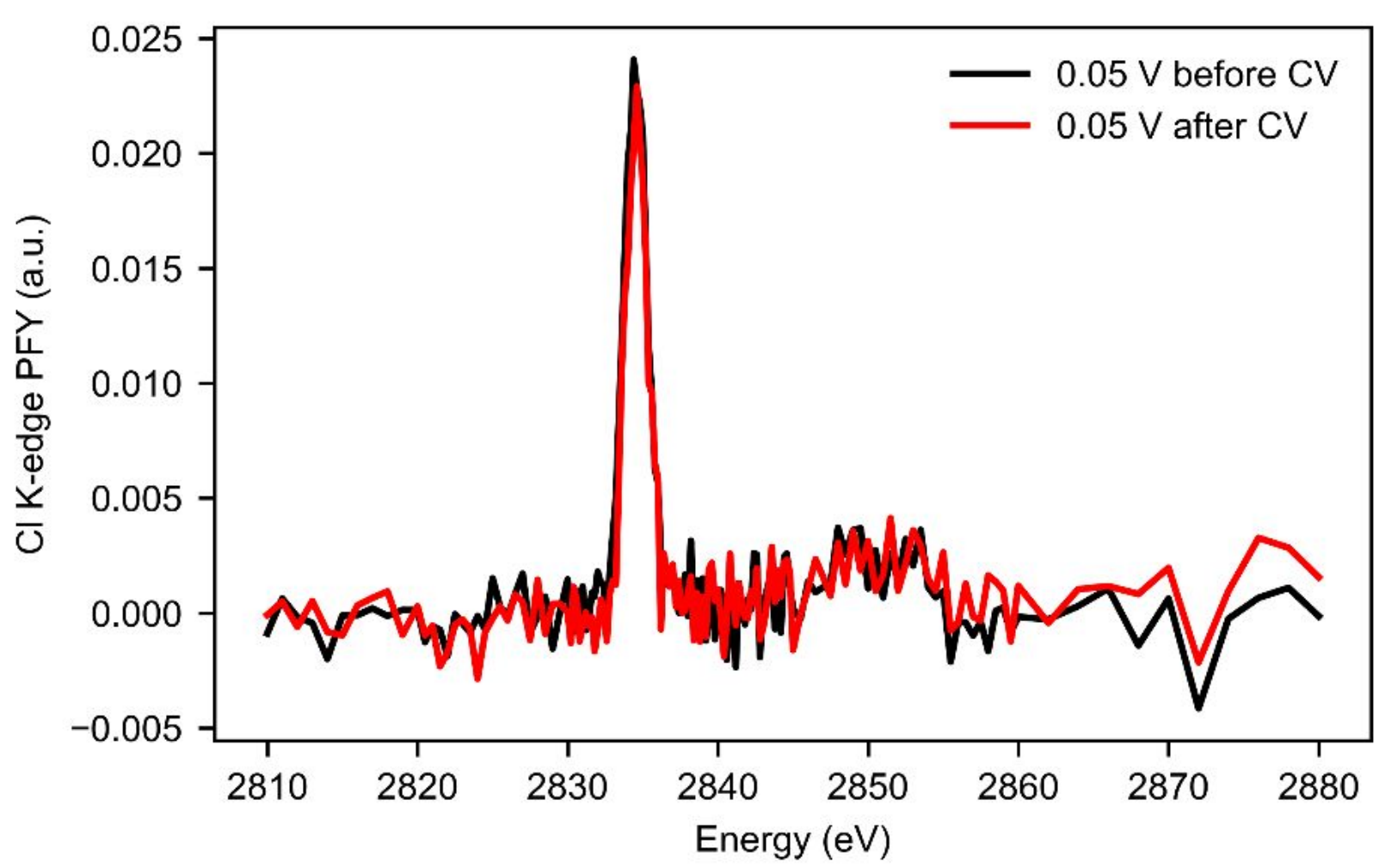

Figure S15: Cl K edge spectra before and after CV showing no change in spectral shape, which illustrates that perchlorate does not decompose due to exposure to the X-ray beam.

12: Time-resolved XRF signal

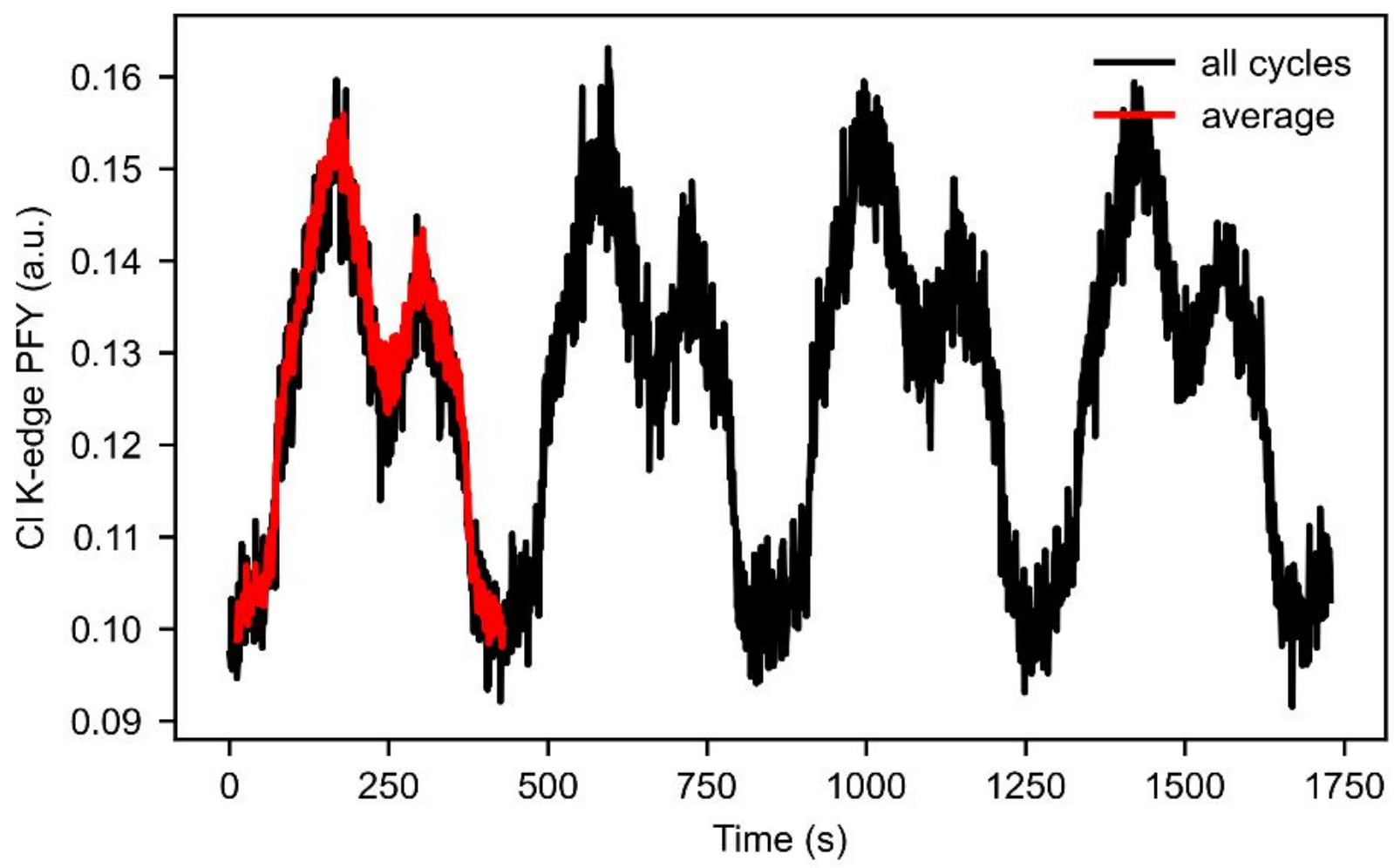

Figure S16: Time-resolved Cl Kα XRF signal in resonance with the white line of the Cl K-edge.

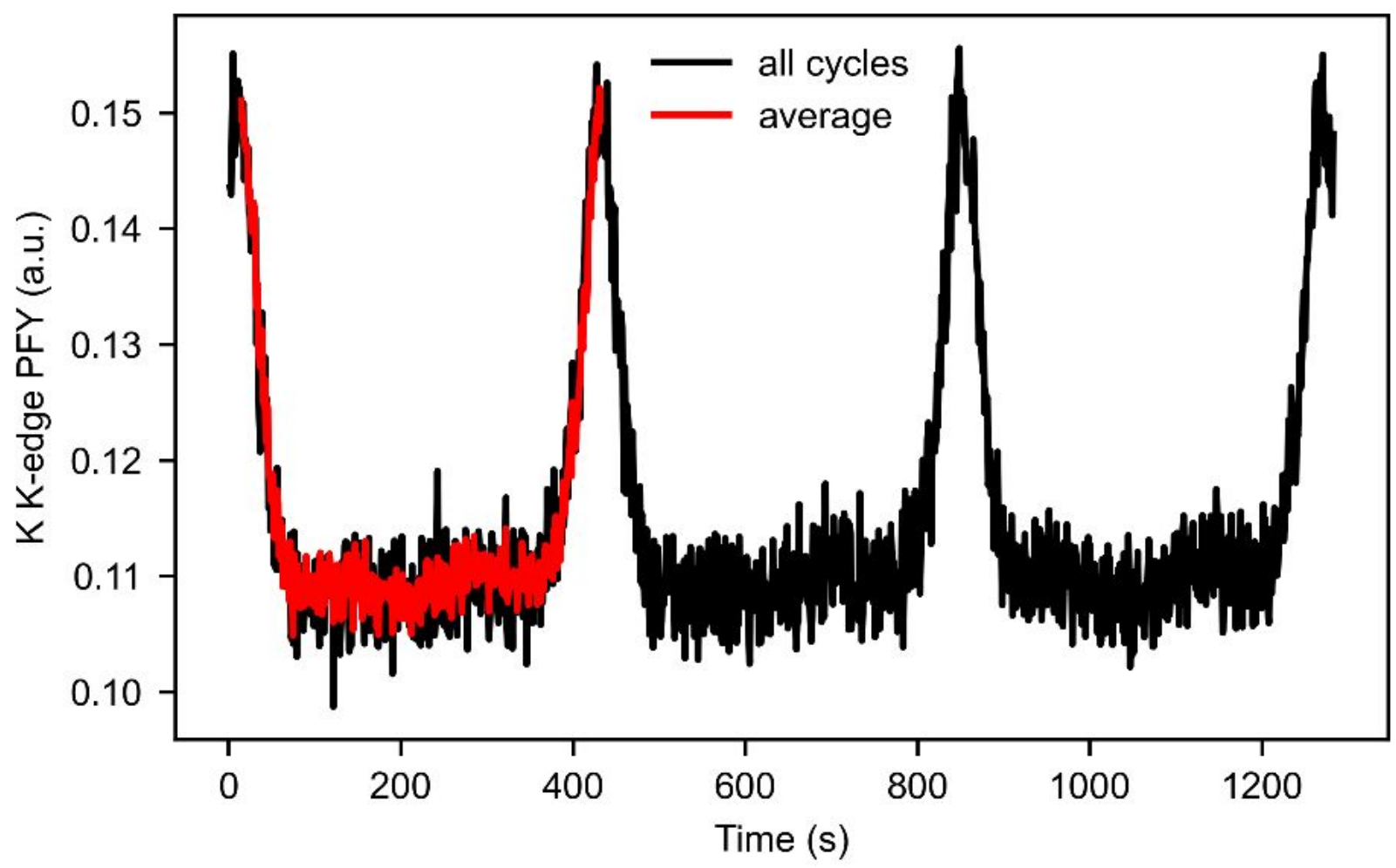

Figure S17: Time-resolved K Kα XRF signal in resonance with the white line of the K K-edge.

13: Bulk signal subtraction and data processing

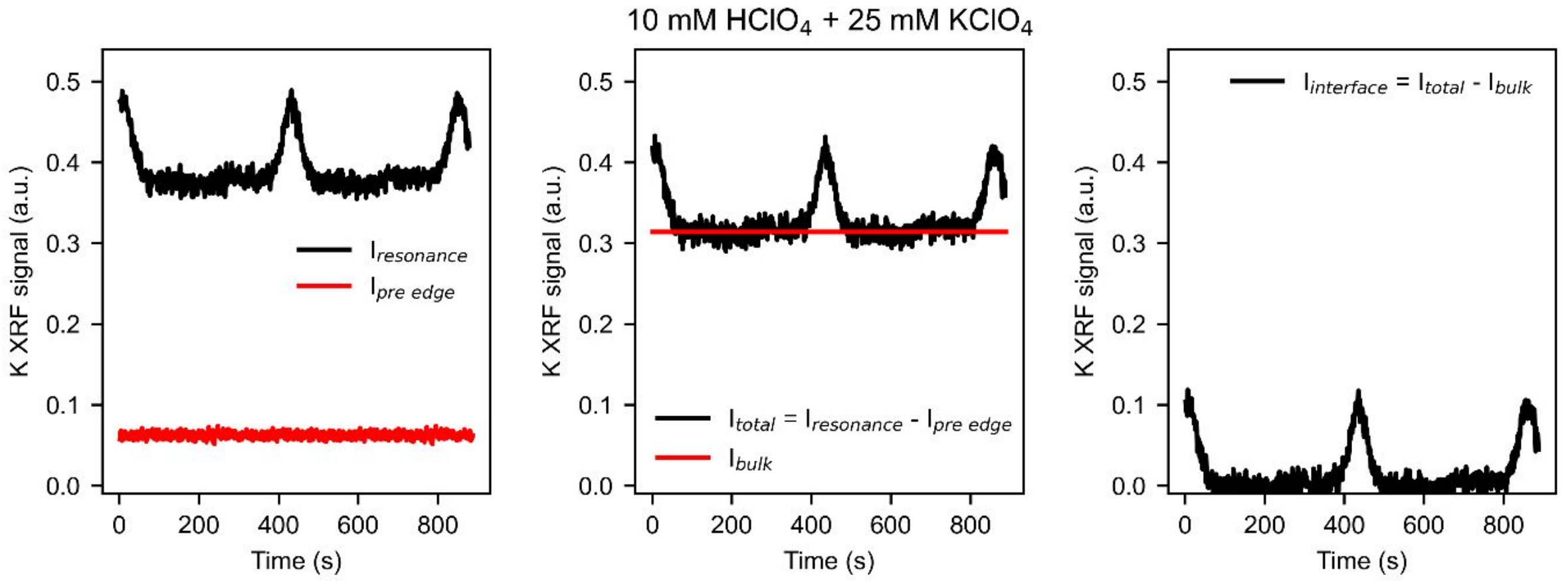

Figure S18: Background subtraction procedure for K XRF data in pH 2 $HClO_4$ + 25 mM K^+

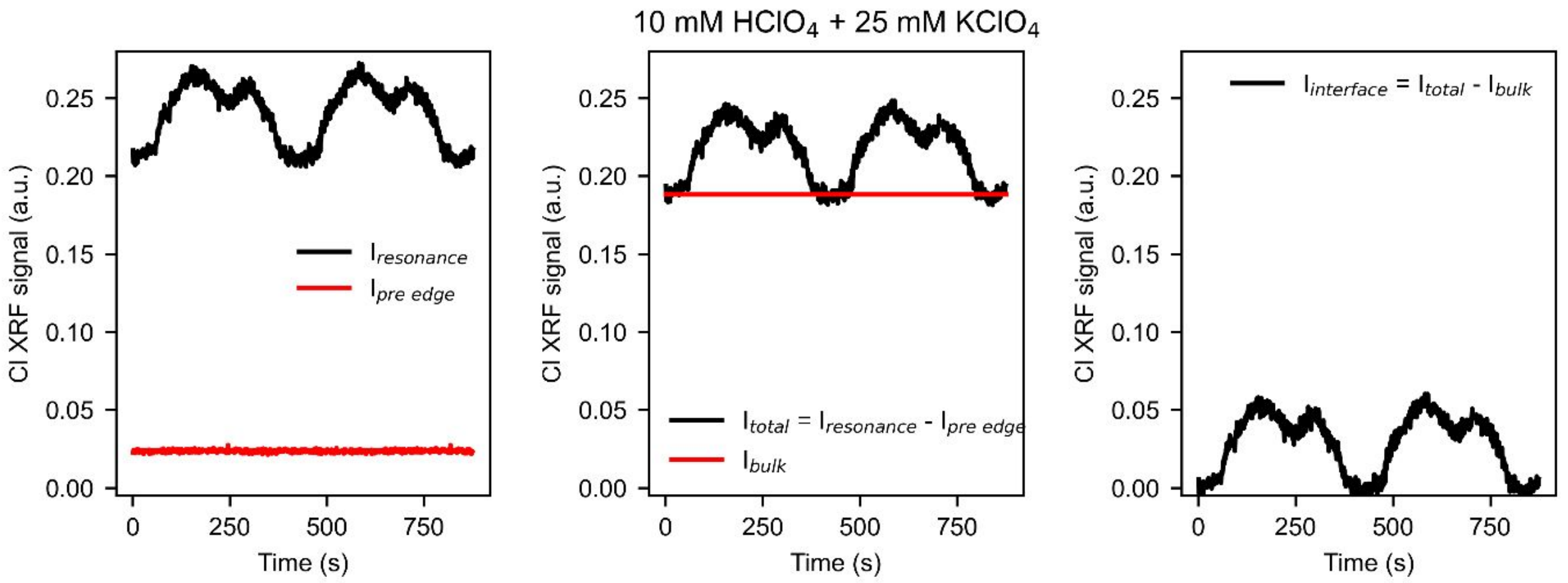

Figure S19: Background subtraction procedure for Cl XRF data in pH 2 $HClO_4$ + 25 mM K^+

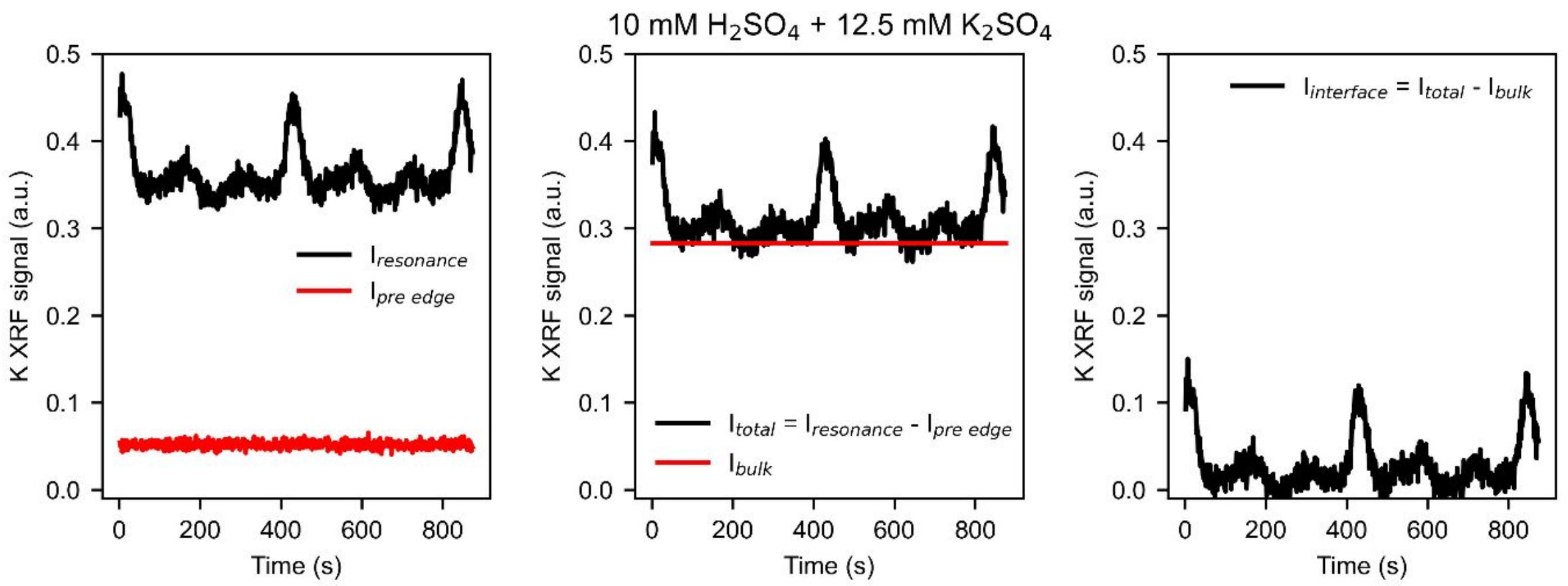

Figure S20: Background subtraction procedure for K XRF data in pH 2 H_2SO_4 + 25 mM K^+

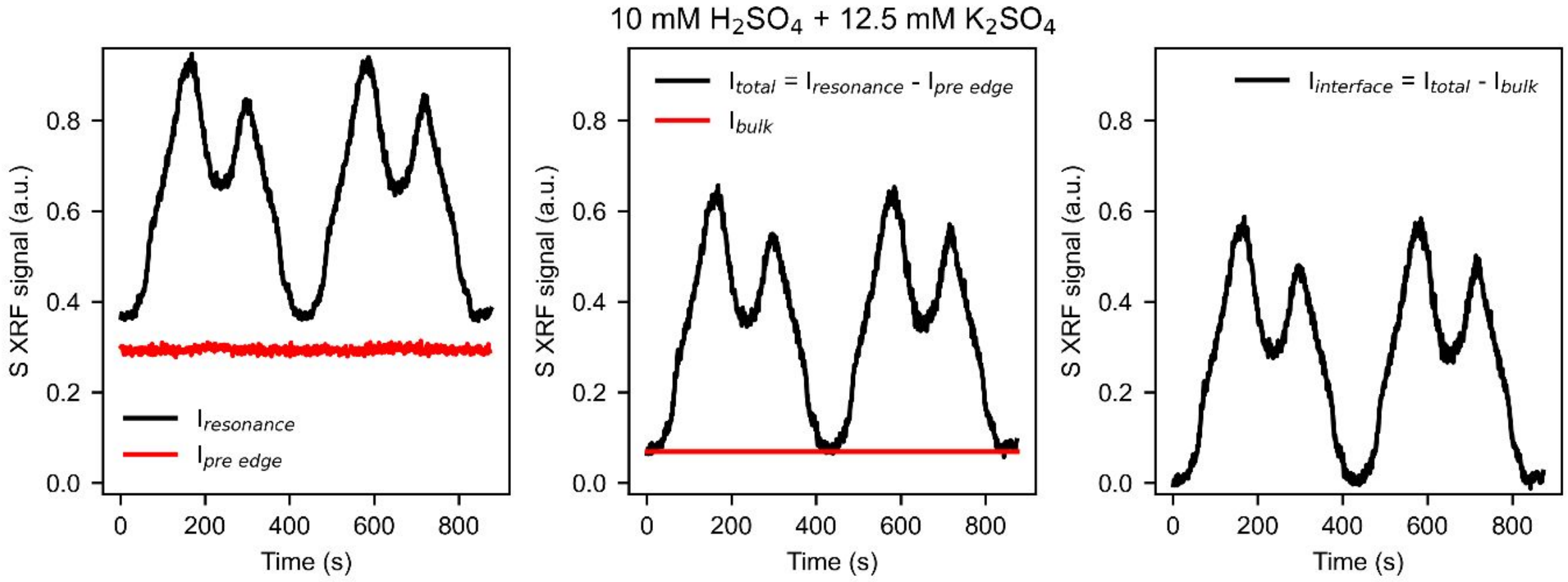

Figure S21: Background subtraction procedure for S XRF data in pH 2 H_2SO_4 + 25 mM K^+

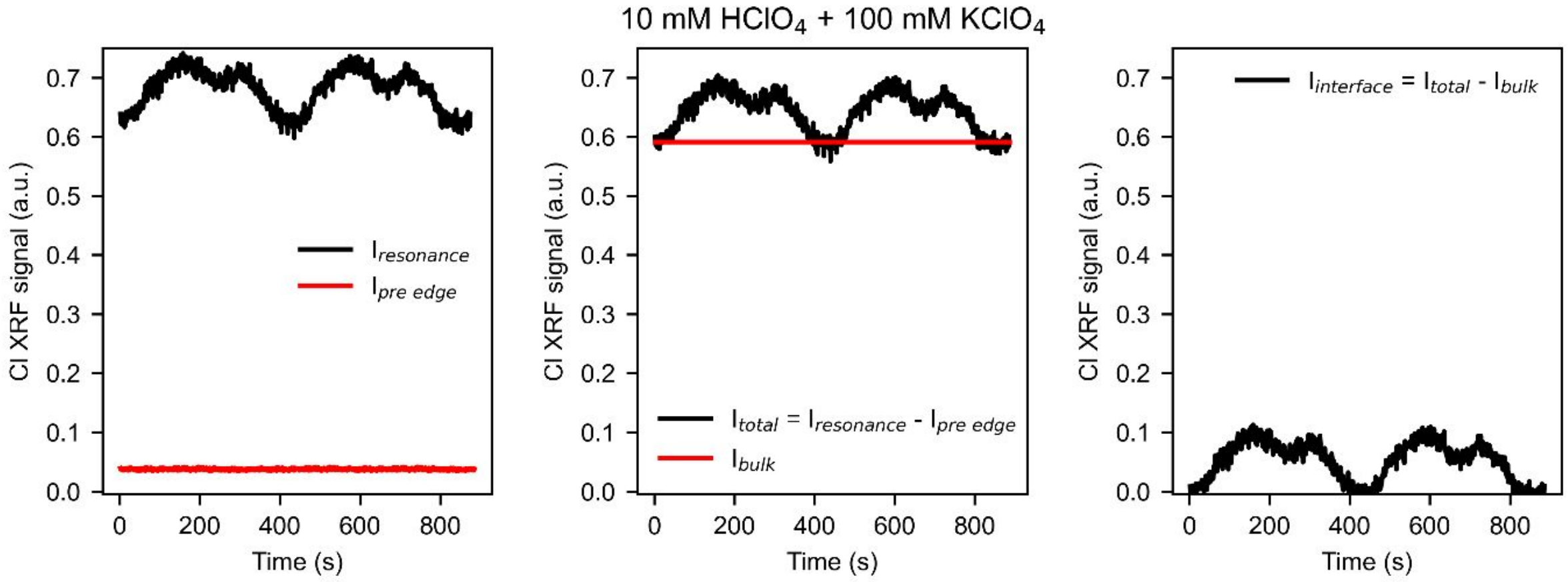

Figure S22: Background subtraction procedure for K XRF data in pH 2 $HClO_4$ + 100 mM K^+

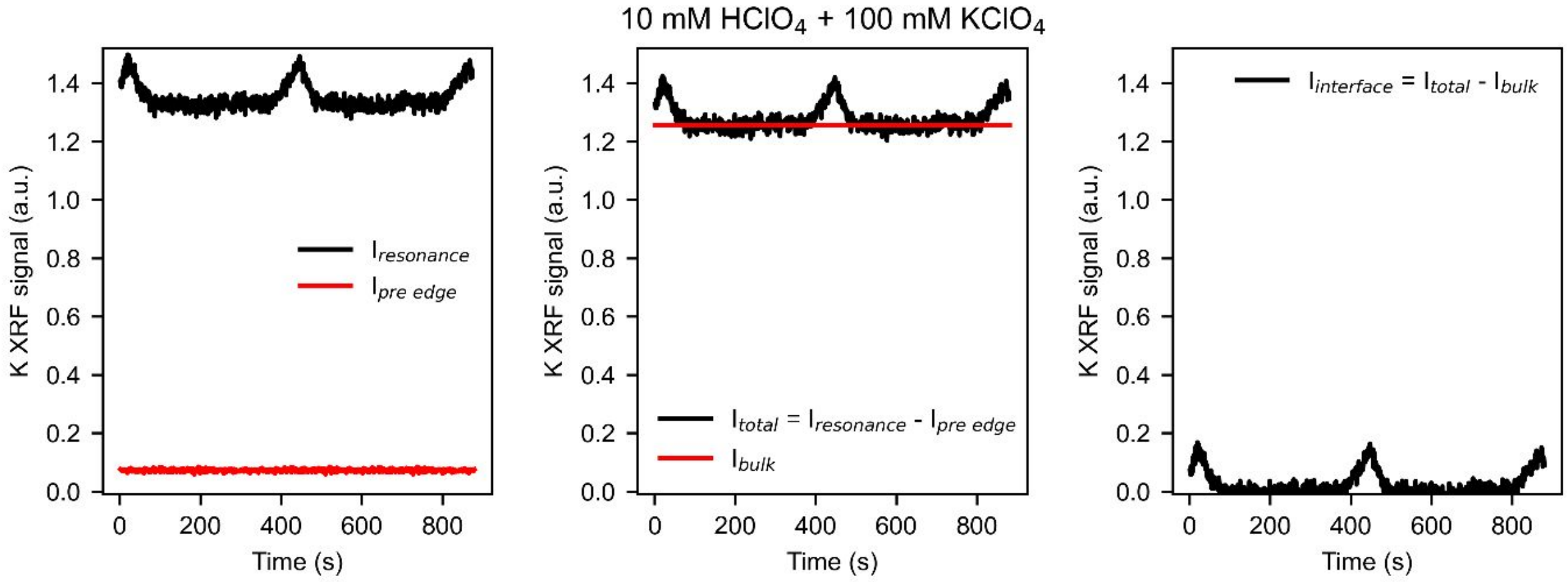

Figure S23: Background subtraction procedure for Cl XRF data in pH 2 $HClO_4$ + 100 mM K^+

Figures S18-S23 show the pre edge and bulk subtraction procedure for the sulfate and perchlorate electrolytes containing K^+. For each time trace, an individual measurement at an energy lower than that of the respective K-edge (pre-edge) was measured. Note that the pre-edge signal shows no time and potential dependence, which is important for a reliable subtraction. The pre-edge intensity was averaged and subtracted from the on-resonance XRF intensity to obtain the total ion signal (bulk + interface). The bulk signal was then defined for each time trace and each electrolyte as the potential range where the ion signal was at its

minimum and unresponsive to the applied electrode potential. For K^+ in the perchlorate electrolyte, this is straightforward, as a large flat region is observed for potentials above the PZFC. This is also in line with previous observations from radioactive tracer experiments for Na[1] and our own EQCM measurements (Figure S5), which show overlapping curves above the PZFC in the presence/absence of alkali cations, implying that they are fully absent in the EDL. For S (SO_4^{2-}), it is also straightforward to define a potential window where the ion signal is not responsive to the applied potential below the PZCF. This also goes in line with previous radioactive tracer experiments [2]. Following this logic, we define the potential range for the perchlorate bulk intensity in the same way. For the K^+ in sulfate-containing electrolytes, where cations and anions coexist, the situation is more complicated. Here, we make use of the EQCM data, shown in Figure S5, where the curves for sulfuric acid with and without Cs^+ ions (a heavy cation was chosen to achieve high mass-sensitivity with the EQCM) overlap at high potentials where the surface oxidizes and the sulfate-electrode interaction becomes weaker, causing the overscreening that attracts K^+ to vanish. The same is observed for other specifically adsorbing anions that also lead to cation accumulation, such as Cl and Br, as shown by radioactive tracer experiments[1]. We can therefore define the highest potential, where we observe the lowest K signal, as the potential range over which we measure only the bulk K signal in the sulfate-containing electrolyte.

The rigor of the analysis procedure and the quality of the dataset become evident when comparing Figure S18 with S22, K data in pH 2 $HClO_4$ + 25 mM K^+ and + 100 mM K^+, respectively. The pre-edge signal does not vary significantly between the two measurements in the two different electrolytes, even though these were recorded on different days during the beam time. This provides an indication of the stability of the beam and system alignment. Note also that the bulk ion signal scales accurately with the difference in bulk ion concentration, 25 mM vs 100 mM. The same is true for the perchlorate signal in Figures S19 and S23. This linear scaling with bulk concentration shows that the XRF sensitivity did not vary significantly throughout the experimental series, which is crucial for quantitative comparison of interfacial ion signals in different electrolytes.

References

1. Kazarinov, V.E., O.A. Petrii, V.V. Topolev, and A.V. Losev, *Potenial Dependence of the Adsorption of Sodium Cations on Platinum and Rhodium Electrodes in Acidic Solutions.* Sov. Electrochem, 1971. **7**: p. 1321.
2. Petrii, O.A. and S. D.A, *Adsorption of Sulfuric-Acid Anions on Platinized Platinum from 1 N H2SO4.* Sov. Electrochem., 1976. **12**: p. 912.
